# Statistical prediction of protein structural, localization and functional properties by the analysis of its fragment mass distributions after proteolytic cleavage

**DOI:** 10.1038/srep22286

**Published:** 2016-02-29

**Authors:** Mikhail I. Bogachev, Airat R. Kayumov, Oleg A. Markelov, Armin Bunde

**Affiliations:** 1 Biomedical Engineering Research Center, St. Petersburg Electrotechnical University, St. Petersburg, 197376, Russia; 2 Department of Genetics, Institute of Fundamental Medicine and Biology, Kazan (Volga Region) Federal University, Kazan, Tatarstan, 420008, Russia; 3 Institut für Theoretische Physik, Justus-Liebig-Universität Giessen, 35392 Giessen, Germany

## Abstract

Structural, localization and functional properties of unknown proteins are often being predicted from their primary polypeptide chains using sequence alignment with already characterized proteins and consequent molecular modeling. Here we suggest an approach to predict various structural and structure-associated properties of proteins directly from the mass distributions of their proteolytic cleavage fragments. For amino-acid-specific cleavages, the distributions of fragment masses are determined by the distributions of inter-amino-acid intervals in the protein, that in turn apparently reflect its structural and structure-related features. Large-scale computer simulations revealed that for transmembrane proteins, either *α*-helical or *β* -barrel secondary structure could be predicted with about 90% accuracy after thermolysin cleavage. Moreover, 3/4 intrinsically disordered proteins could be correctly distinguished from proteins with fixed three-dimensional structure belonging to all four SCOP structural classes by combining 3–4 different cleavages. Additionally, in some cases the protein cellular localization (cytosolic or membrane-associated) and its host organism (*Firmicute* or *Proteobacteria*) could be predicted with around 80% accuracy. In contrast to cytosolic proteins, for membrane-associated proteins exhibiting specific structural conformations, their monotopic or transmembrane localization and functional group (ATP-binding, transporters, sensors and so on) could be also predicted with high accuracy and particular robustness against missing cleavages.

In the last decades, due to the intensive development of omics technologies, there is an increasing demand in predicting the structural and structure-related properties of biological polymers. While several experimental methods to uncover the structure of biological molecules such as X-ray crystallography, nuclear magnetic resonance and cryo-electron microscopy have been established, they still appear expensive and time-consuming and thus can cover a relatively small part of the rising number of laboratory research activities. Therefore the structural characterization of biological polymers like proteins and RNA is largely based on reconstructions from the arrangement of monomers in their primary sequences.

An important step in the statistical analysis of biomolecular structures was the discovery of pronounced long-range correlations (LRC) in the DNA primary sequences in the early 90s^1^,^2^,^3^,^4^,^5^. Grossberg and coauthors has attributed the existence of LRC to the hierarchical three-dimensional architecture of DNA^6^. In the following, the LRC has been shown to additionally reflect several physical, structural and functional properties of biological polymers including the DNA bending profile^7^,^8^,^9^,^10^,^11^. Very recently we have shown that the LRC is explicitly reflected in the distributions and in the persistence properties of the intervals between the same nucleotides in the DNA primary sequence. In particular, we found that the interval distributions follow a universal single *q*-exponential law in both pro- and eukaryotes at relatively small scales, while at large scales a second additive *q*-exponential term with the same *q* can be observed specifically in eukaryotes^12^.

While the double-helix DNA structure is irrelevant to the particular order of nucleotides^13^ leading to the universal statistical laws governing their arrangement in the primary sequence^12^, the arrangement of amino acid residues in a polypeptide chain defines its folding via chain bending and hydrogen bonds formation^14^,^15^,^16^. In particular, when properly arranged, such amino acids as methionine (M), alanine (A), leucine (L), glutamate (E) and lysine (K) are mainly involved in the formation of three-dimensional helical conformations known as *α*-helixes. Proline (P) and glycine (G) have unusual conformational abilities and disrupt the regularity of the *α*-helical backbone conformations. The large aromatic residues such as tryptophan (W), tyrosine (Y) and phenylalanine (F) as well as the branched-chain amino acids including isoleucine (I), valine (V), and threonine (T) prefer to adopt *β*-strand (or *β*-sheet) conformations. Most proteins contain both *α*-helixes and *β*-sheets, and the way of their alternation along the polypeptide chain and bending determines the tertiary and the quaternary structures of the functional protein. Based on the relative fractions of *α*-helixes and *β*-sheets as well as the ways of their spatial orientation a common structural classification of proteins (SCOP) notation has been introduced in^17^. It includes four main structural classes, namely all-*α*, all-*β*, *α*/*β* and *α* + *β* classes (for a comprehensive description see, e.g.^18^, and references therein). Once the primary polypeptide chain is available, both the secondary and the tertiary protein structures can be reconstructed with high accuracy using existing molecular modeling algorithms^19^,^20^. Examples of such reconstructions using the Phyre2 algorithm^20^ are shown in Fig. 1 for two sample transmembrane proteins, namely the cytochrome B that spans the mitochondrial membrane with 8 transmembrane *α*-helixes^21^ (Fig. 1(a)), and the maltoporin-like channel (LamB porin) forming a trimeric structure that facilitates the diffusion of maltodextrins and other sugars across the outer membrane of Gram-negative bacteria formed by 18 antiparallel *β*-strands^22^ (Fig. 1(b)).

Due to the significant improvements in the computational protein structure prediction methods over the last two decades, extraction of structural information from protein primary polypeptide chains is now much less challenging. Starting in the early 90s with somewhat 70–75% secondary structure prediction accuracy^23^,^24^, these methods soon became capable of predicting particular protein structural class from its given primary sequence with 80–90% accuracy at least for highly homologous datasets^25^ and with well above 90% accuracy for special cases such as membrane proteins^26^,^27^. Classification of protein groups with considerably lower (25–40%) homology remained more challenging^18^,^28^,^29^, while recent investigations seem to have successfully overcome the 80% accuracy barrier even for the low homology datasets^30^,^31^. A number of mathematical approaches has been utilized, including neural networks^23^, scoring matrices^24^, RoughSets-based algorithms^25^, component-coupled methods^28^, collocation-based structural representation^29^, pseudo-amino-acid structural encoding^31^ and some others. Most of the suggested algorithms require considerable computational efforts including calculation of several dozens of parameters for each primary polypeptide chain and creating multi-parametric statistical classification models based on either support vector machines, instance-based, information discrepancy and geometric classifiers, logistic regression or Bayessian classifiers (see, e.g.^18^, and references therein). Recently the amino acid distribution along the polypeptide chain assessed by correlation analysis has been suggested as a powerful tool of protein bending prediction^32^.

However all above listed approaches require the primary polypeptide chain of the studied protein to be available. Primary polypeptide chains of proteins are usually obtained by translation of the coding parts of the primary DNA sequences that in turn requires a sequenced and annotated genome of the studied organism to be available. In recent years a powerful alternative has been provided by the so-called *de novo* protein sequencing that is based on the reconstruction of the protein polypeptide chain from its high-throughput mass spectrometry data with subsequent sequence reconstruction using statistical models^33^,^34^,^35^,^36^,^37^,^38^. Despite its recent success, *de novo* sequencing is currently not widely practiced for protein analysis and identification, since high-throughput equipment is required, while problems of low resolution and incomplete fragmentation persist leading to inevitable sequencing errors that occur in about 70–75% of assembled polypeptide chains^34^. Since a considerable fraction of sequencing errors will likely disrupt the performance of sequence analysis methods leading to a breakdown in the protein structure prediction accuracy, they are rather more suitable for the analysis of identified proteins with accurately determined sequences.

A more common and relatively affordable way to identify proteins in laboratory conditions is based on the analysis of the masses of polypeptide chain fragments after its amino acid-specific proteolytic cleavage. These masses are assessed by the matrix-assisted laser desorption/ionization (MALDI) time-of-flight (TOF) spectrometry, a sophisticated technique allowing to determine the oligopeptide molecular masses with high accuracy and this way providing explicit information on their content, but not on the arrangement of amino acid residues in the polypeptide chain. The identification is based on the comparison of the experimental mass spectrum with the theoretical mass spectra obtained by a software simulation of the amino acid specific cleavage of known proteins from a database (exemplified in Fig. 2). The protein is then identified by the minimal distance between the experimental and the theoretical mass spectra using conventional methods to compare spectra of highly homologous biomolecules^39^.

The question is how to proceed when no highly homologous proteins are found in the database, or different fragments of the studied protein show comparably high alignment scores with different proteins in the database, a common situation for multi-domain proteins, and thus no direct conclusions can be drawn from the spectra comparison. To partially overcome this problem, in this paper we suggest an approach to predict several structure-associated properties of arbitrary proteins directly from the mass distributions of their proteolytic cleavage fragments without reconstruction of their primary polypeptide chains. As we show below, in some cases this leads also to the successful prediction of cellular localization and functional group of the studied protein. The theoretical background of our approach is based on the analysis of the distributions of inter-amino-acid intervals in the primary polypeptide chain. On one hand, the interval distributions explicitly reflect the structural organization of biological polymers, as we have shown recently for the DNA^12^ and here confirm also for several protein classes. On the other hand, intervals between those amino acids that specify cleavage sites directly determine the sizes and thus also the masses of polypeptide chain fragments after cleavage. Accordingly we suggest that the statistical laws governing the amino acid arrangement should be explicitly reflected in the mass distributions of the post-cleavage oligopeptides.

The paper is organized as follows. First, we introduce the major quantities of interest, namely the exceedance probability distributions of both the inter-amino-acid intervals and the oligopeptide masses. As a null hypothesis, we consider the (hypothetical) random allocation of amino acid residues along the polypeptide chain. In this case, the distribution of intervals between the consecutive positions of any given amino acid residue is theoretically expected to decay by a simple exponential, leading to the same asymptotic behaviour of the post-cleavage mass distribution. Therefore we focus our prediction strategy on the analysis of the deviations of the empirical exceedance probability distributions from the theoretical null hypothesis (exponential) distribution, this way emphasizing the (presumably) structure-associated non-random arrangement of the amino acids in the primary polypeptide chain. To quantify these deviations, we focus on the maximum discrepancy between the empirical and the theoretical (null hypothesis) exponential distributions, a quantity that is used in the widely adopted Kolmogorov statistical test. Based on this quantity, we next suggest a simple prediction model design. To further improve the prediction accuracy, we subsequently combine the (potentially complementary) statistics for different cleavage rules into a single logistic regression model. The overall performance of the prediction model is then evaluated by receiver operator characteristic (ROC) analysis^40^. For deeper details on the theoretical background of our approach as well as the details of the statistical analysis, we refer to the Methods section at the end of this paper.

For a detailed stepwise demonstration of the entire analysis including the decision-making procedure, we focus on two prominent examples where binary classifications are suitable. In particular, we consider in detail (i) the prediction of transmembrane proteins carrying either *α*-helixes or *β*-sheets in their secondary structure as well as (ii) the prediction of membrane-associated proteins from either *Firmicutes* or *Proteobacteria* according to their host bacteria phylum. We show explicitly that the secondary structural classification of transmembrane proteins could be correctly performed in approximately 90% cases by using a single cleavage rule, while proteins with specific localization in bacterial cell membranes could be correctly predicted as belonging to either *Firmicutes* or *Proteobacteria* in more than 80% cases by combining three to four different cleavages.

In the following, to test the potential applicability of our approach to predict a number of structural, localization and functional properties of a given protein, we perform a large-scale validation analysis using various protein groups obtained from the general proteomic databases UniProtKB/Swiss-Prot^41^ and RSCB PDB^42^, from the databases of intrinsically disordered proteins DisProt^43^ and Ideal^44^ as well as from the transmembrane protein database PDBTM^45^ and the bacterial virulence factors database VFPB^46^. We employ simple logistic regression models for all binary classification criteria and perform discriminant analysis for all multiple classification criteria. To facilitate cross-validation, in all cases we first randomly select about 70% of proteins from each group and use them to fit the model parameters, subsequently performing tests on both (70%) selected and (30%) unselected groups. Results of large-scale computer simulations (summarized in the Supplementary information available online) reveal that many structural and localization properties of proteins can be predicted with 75–80% accuracy by combining 1–3 different cleavages. Remarkably, we could also correctly predict about 3 out of 4 intrinsically disordered proteins within low-homology datasets that additionally included proteins from all four SCOP structural classes.

Finally, to further emphasize the simplicity of our approach and to illustrate the simple validation model design, we provide two test samples for the ammonium transporter AmtB and glutamine synthetase proteins from *B.subtilis* (as Supplementary datasets 1 and 2 available online) where the entire procedure from the analysis of post-cleavage oligopeptide masses (obtained from^47^) until the prediction of structural, localization and functional properties are implemented as spreadsheets. For testing purposes, one can simply replace the oligopeptide masses list with one from an arbitrary protein and see whether it fits.

## Results and Discussion

### Predicting structural classes of transmembrane proteins

#### Inter amino acid interval distributions

In the last decades, many investigations confirmed that a complex secondary structure of biological polymers is reflected in the distribution pattern of monomers in their primary sequences (for an extensive review, we refer to^7^). Recently we have shown that the distribution of inter-nucleotide intervals in DNA reflects its packaging in eukaryotes^12^. Since the protein sequences are much shorter compared to the DNA, it appears much more difficult to determine particular functional forms of the interval distributions between amino acid residues for single proteins. Therefore we first analyzed the interval distributions of amino acids for an entire group of proteins with a given property. We consider the exceedance probability distributions *R*(*l*) defined as the probability that the interval exceeds a given value *l* as a function of *l* (for more details on the interval distributions and their interpretation, we refer to the Methods section at the end of this paper).

Figure 3 shows the exceedance probability distributions *R*(*l*) for the intervals between amino acids in 5904 transmembrane proteins from pro- and eukaryotes with different secondary structures forming either *α*-helixes or *β*-sheets (obtained from^45^). To improve the statistics, instead of showing the interval distributions for each protein, the overall *R*(*l*) for the considered protein group is shown.

The figure shows that with an exception of a single amino acid residue C (cytosine), for other 19 amino acids heavy-tailed interval distributions (that decay considerably slower than an exponential) appear a specific feature of *α*-helixes that cannot be observed in *β*-sheets. This finding further supports the relationship between the interval distribution and the structural complexity of biomolecules that we have recently discovered in DNA^12^. As a control, the amino acid residues in both protein groups were randomly shuffled and the exceedance probability distributions of the inter-amino-acid intervals were obtained for the shuffled sequence. Figure 3 shows that after shuffling all the interval distributions exhibited universal exponential behaviour. This indicates that the observed differences in the inter-amino-acid interval distributions of *α*-helical and *β*-barrel transmembrane proteins are determined by arrangement of amino acids in their primary sequences.

#### Mass spectrometry simulation

**Figure 2:**
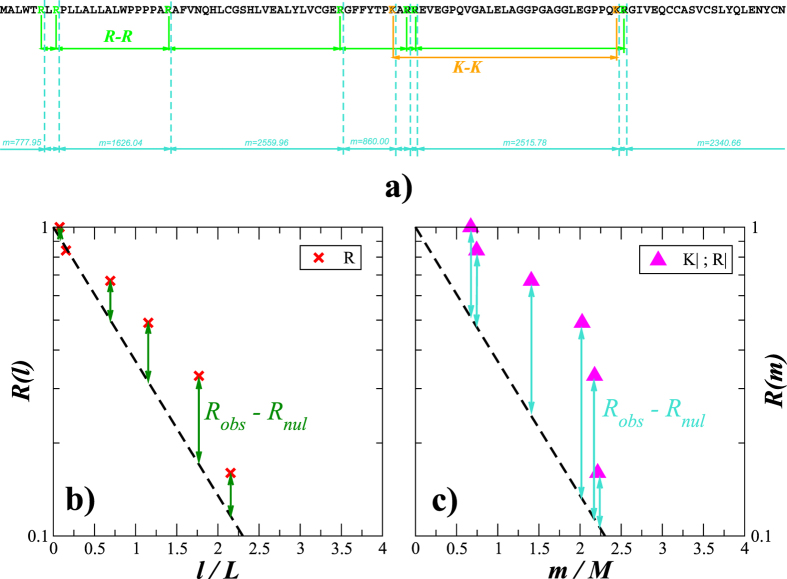
(**a**) Inter-amino-acid intervals and protein cleavage procedure exemplified for the full *B.taurus* insulin sequence. Full vertical lines indicate the positions of two specific amino acids K (orange) and R (green) and intervals between their positions are shown by horizontal lines of the same color. Dashed vertical lines indicate specific cleavage positions by Trypsin (cleavage at C-terminal side of either ‘K’ or ‘R’). Molecular masses of oligopeptides obtained after successful cleavage of the polypeptide chain are annotated at the horizontal lines. (**b**) Exceedance probability distribution functions 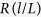 of the inter-amino-acid intervals and (**c**) 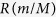 of the oligopeptide masses for the same sample protein in the units of average intervals *L* and average masses *M*, respectively. Arrows show the distances 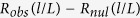 and 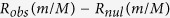 between the observational and the theoretical exponentially decaying exceedance probability distribution functions 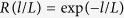 and 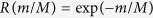 (shown by dashed lines) representing the null hypothesis (random allocation of amino acids) for the interval and the mass distributions, respectively.

**Figure 3:**
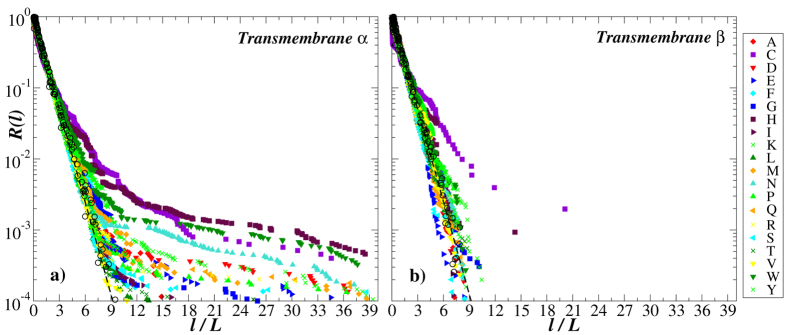
The exceedance probability distributions of intervals between amino acids in transmembrane proteins carrying presumably (**a**) *α*-helixes and (**b**) *β*-sheets in their secondary structure. For comparison, the open black circles show the same distributions for shuffled amino acid sequences, while the dashed line shows a simple exponential 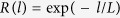.

Nowadays, identification of unknown proteins is commonly based on their amino acid-specific proteolytic cleavage followed by the comparison of the experimental molecular mass spectrum of the obtained cleavage fragments against the simulated mass spectra of the same cleavage of all proteins in the given database. Since the oligopeptide sizes are directly determined by the interval distributions between the amino acids that specify cleavage sites (see Fig. 2), and the functional form of the interval distributions between amino acid residues reflects the proteins structural complexity, we asked whether the knowledge of the distribution pattern of oligopeptide masses after protein cleavage could be used to predict various structural and structure-associated properties of the analyzed protein.

**Figure 4:**
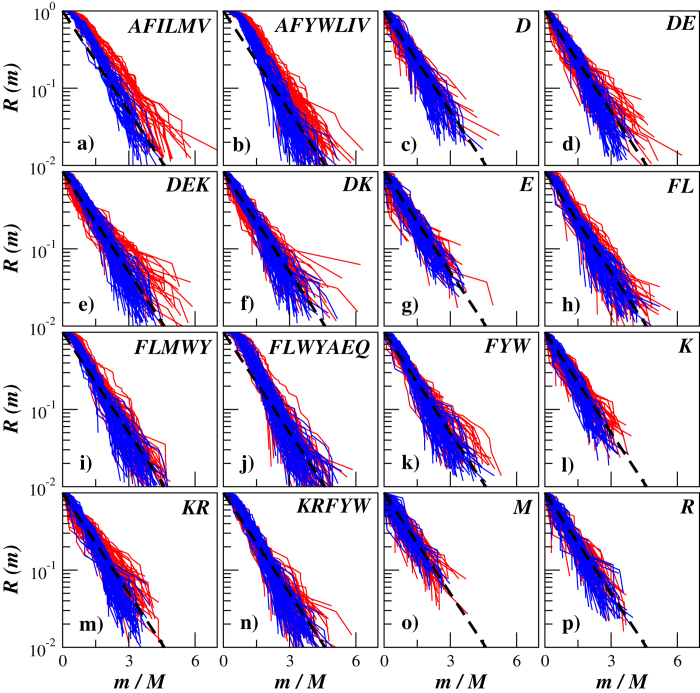
The exceedance probability distributions of oligopeptide masses in transmembrane proteins carrying either *α*-helixes (red) or *β*-sheets (blue) in their secondary structure exemplified for 100 randomly selected proteins from each group. For comparison, the dashed lines show simple exponentials 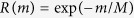.

Figure 4 shows the exceedance probability distributions of oligopeptide masses in transmembrane proteins carrying either *α*-helixes or *β*-sheets in their secondary structure exemplified for 100 randomly selected proteins from each group and for 16 different cleavage rules that can be implemented using commercially available enzymes/reagents (for a detailed description on available enzymes/reagents leading to different cleavage rules as well as relevant exceptions from them we refer to the resource portal of the Swiss Institute of Bioinformatics^48^).

The figure shows that the manifestation of the protein secondary structure is especially pronounced in the mass distributions for those cleavage rules that include amino acid residues involved in the formation of *α*-helixes and *β*-sheets as cleavage sites. In particular, the distributions of oligopeptide masses after simulated cleavage by Thermolysin that includes alanine (A), leucine (L) and methionine (M) involved in the formation of *α*-helixes as well as phenylalanine (F), isoleucine (I) and valine (V) involved in the formation of *β*-sheets as cleavage sites appear considerably broader in proteins carrying *α*-helixes in their secondary structure (see Fig. 4(a)). Remarkably, five out of six amino acids that specify cleavage sites by Thermolysin belong to the inertial hydropathy group. Analysis of amino acid residues arrangement in the polypeptide chain according to their hydropathy profile has been successfully used in the sequence-based protein structural class prediction methods including recent top-performance methods^31^. A very close situation can be observed in the mass distributions after simulated cleavage by Proteinase K, that also includes alanine (A) and leucine (L) involved in the formation of *α*-helixes as well as phenylalanine (F), isoleucine (I), valine (V), tryptophan (W) and tyrosine (Y) involved in the formation of *β*-sheets as cleavage sites (see Fig. 4(b)). Similar but slightly weaker effects can be observed for cleavages by Chymotrypsin and by Pepsin that include leucine (L) and methionine (M) involved in the formation of *α*-helixes as well as phenylalanine (F), tryptophan (W) and tyrosine (Y) involved in the formation of *β*-sheets as cleavage sites (see Fig. 4(h,i)). This effect could be attributed either to the more complex arrangement of amino acid residues involved in the formation of *α*-helixes reflected in the broad oligopeptide mass distributions, or to the periodic alternation of amino acid residues involved in the formation of *β*-sheets as well as to the possible combination of these effects. In contrast, for many other cleavage rules the distributions either differ slightly or appear rather similar in both studied protein groups.

#### Statistical analysis and classification

For a quantitative assessment of the heavy tails in the oligopeptide mass distributions, we consider the standard Kolmogorov statistics using a simple exponential distribution that corresponds to the (hypothetical) random arrangement of the amino acid residues in the primary polypeptide chain as a theoretical null hypothesis. In the Kolmogorov statistical test one considers the maximum difference between the observational and the theoretical (null hypothesis) cumulative probability distributions 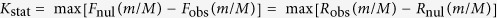 out of all (normalized) oligopeptide masses *m*/*M* obtained for a particular protein (exemplified by vertical arrows in Fig. 2(c)). To test against the null hypothesis 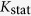 is usually compared against a threshold value that depends on the number of observed masses and on the significance level.

**Figure 5:**
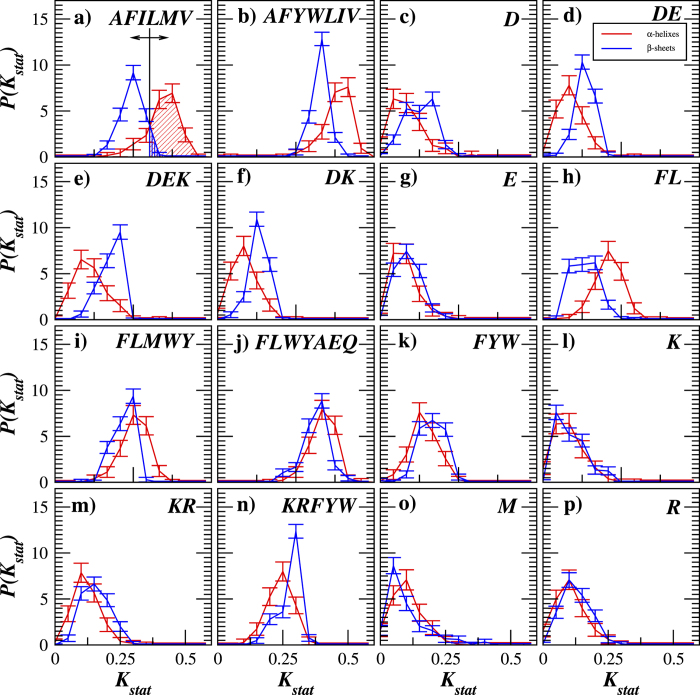
The empirical probability distribution function (PDF) estimates of *K*_stat_ values in transmembrane proteins carrying either *α*-helixes (red) or *β*-sheets (blue) in their secondary structure. The black vertical line in (**a**) indicates a variable decision threshold 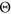 used to calculate a ROC-curve, while the diagonal and the vertical filled areas denote the hit rate and the false alarm rate for the chosen threshold 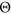, respectively.

Figure 5 confirms that 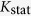 distributions vary between the proteins with *α*-helixes and *β*-sheets in their secondary structure exhibiting the smallest overlap for the Thermolysin (AFILMV) and Proteinase K (FLWYAEQ) cleavages (see Fig. 5(a,b)). To facilitate the prediction of the protein structure, we next compare 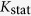 against an arbitrary decision threshold value 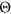 (exemplified by a vertical line in Fig. 5(a)). If 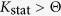, we predict that the protein carries mainly *α*-helixes in its secondary structure, otherwise, we predict that its secondary structure is dominantly formed by *β*-sheets. While this rule provides a correct classification for most analyzed proteins, there are inevitable misclassifications. For an explicit quantification of the prediction accuracy, for any given threshold value 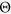 one can calculate the fraction of correctly predicted *α*-helical proteins also known as the hit rate (shown by the diagonal filling in Fig. 5(a)) as well as the fraction of *β*-sheet proteins falsely predicted as *α*-helical proteins also known as the false alarm rate (shown by the vertical filling in Fig. 5a). By varying the decision threshold 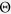, one can optimize it according to a given criteria such as maximum overall accuracy, maximum tolerable false alarm rate and so on. For an overall quantification of the predictive power, one can plot the hit rate as a function of the false alarm rate for all possible threshold values 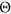 this way obtaining the so-called ROC curve^40^. For deeper details on the ROC curve design and features, we refer to the Methods section of this paper as well as to^40^ and references therein.

**Figure 6:**
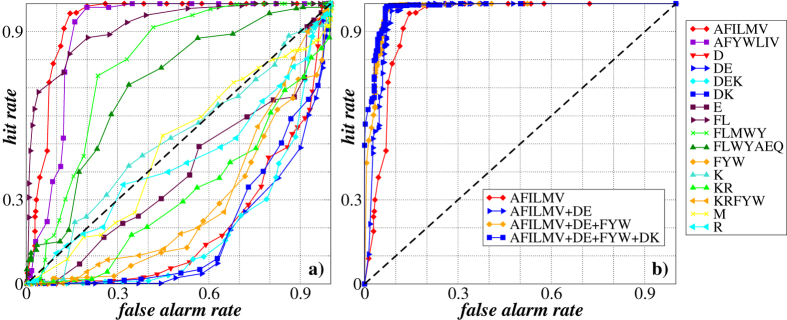
(**a**) The receiver operator characteristic (ROC) curves characterizing the potential prediction accuracy of transmembrane proteins carrying either *α*-helixes or *β*-sheets in their secondary structure according to the oligopeptide mass distributions exemplified for 16 different cleavage rules. (**b**) The ROC curves for the binary logistic regression models representing one to four best linear combinations of predictors using different cleavage rules. The areas under the curves (AUC) are 0.935, 0.961, 0.976 and 0.983, while the Nagelkerke 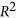 coefficients of determination are 0.727, 0.814, 0.863 and 0.875 for one, two, three and four cleavages used, respectively.

Figure 6(a) shows the ROC curves for each of the 16 single cleavage rules. The diagonal ROC curve states for the case with no predictive power (such as a random guess). In contrast, those ROC curves with maximal deviation from the diagonal that provide high hit rates for low false alarm rates indicate the most powerful predictors. Figure 6(a) shows that the ROC curves are well above the diagonal for those cleavage rules where the 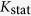 values are considerably greater for the *α*-helical proteins than for the *β*-sheet proteins, in particular for Thermolysin (AFILMV), Pepsin with 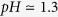 (FL) and Proteinase K (AFYWLIV) (see Fig. 5(a,b,h)). The overall predictor efficacy is then quantified by the area under the curve (AUC)^40^ and reaches 0.94 for the cleavages by Thermolysin (AFILMV), 0.93 by Pepsin with 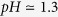 (FL) and 0.90 by Proteinase K (AFYWLIV), respectively.

In contrast, those ROC curves that are well below the diagonal, indicate those cleavages where the 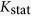 values are typically smaller for the *α*-helical proteins than for the *β*-sheet proteins, like it happens for example for cleavages by GluC(phosphate) (DE), GluC(phosphate)+LysC (DEK) and AspN/LysC (DK) (see Fig. 5(d,e,f)). To employ those predictors, the decision rule has to be inverted, i.e. one should predict an *α*-helical structure protein when 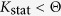 and a *β*-sheet structure protein otherwise. In this case, the area above the curve provides the overall predictor efficacy.

To test whether the prediction accuracy could be further improved by combining the (potentially) complementary cleavage rules, we next design a binary logistic regression model. The model provides an optimized linear combination of 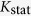 values from several cleavage rules. Those cleavage rules where 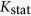 values are greater for the *α*-helical proteins than for the *β*-sheet proteins, are included in the model with a positive sign. In contrast, those cleavage rules where 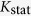 values are smaller for the *α*-helical proteins than for the *β*-sheet proteins, are included in the model with a negative sign. For deeper details on the design of the logistic regression model, we refer to the Methods section of this paper. Next, the resulting linear combination of 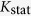 values corresponding to several cleavage rules is also compared with a decision threshold 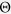, and a ROC curve is obtained, like it has been done for single cleavages.

Figure 6(b) shows the ROC curves for the logistic regression models representing the best linear combinations of one to four cleavage rules. For comparison, the first ROC curve (identical to that one in Fig. 6(a)) corresponds to the single cleavage by Thermolysin (AFILMV). The figure shows that the best combination of two cleavage rules leads to the slight enhancement in the prediction accuracy (AUC = 0.96) while adding further cleavage rules does not lead to significant improvement of the prediction quality any more.

### Predicting cellular localization and host bacteria phylum of proteins from pathogenic *Firmicutes* and *Proteobacteria*

#### Inter-amino-acid interval distributions

Since the secondary and particularly the tertiary structure of proteins are often associated with their localization and function, we next performed a similar analysis of bacterial proteins with different localization and functional role in the cell. We have compared different functional groups of bacterial proteins of pathogenic *Firmicutes* (*Bacilli, Clostridium, Corynebacterium, Staphylococci, Streptococci, Listeria*) and *Proteobacteria* (*Enterobacteriaceae, Pseudomonas, Neisseria, Bartonella, Legionella, Haemophilus, Helicobacter, Yersinia*).

**Figure 7:**
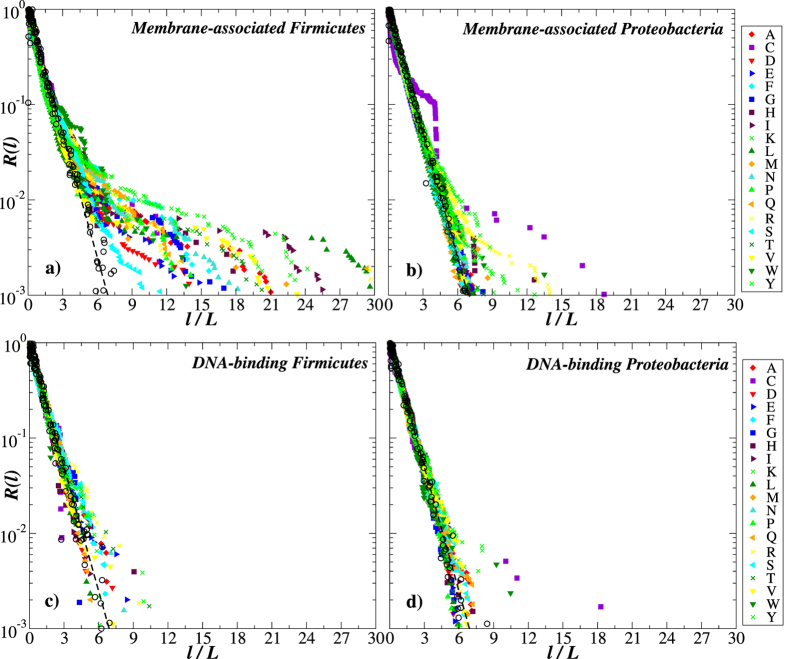
The exceedance probability distributions of the intervals between amino acid residues in (**a**,**b**) membrane-associated and (**c**,**d**) soluble proteins of (**a**,**c**) *Firmicutes* and (**b**,**d**) *Proteobacteria*. For comparison, the open black circles show the same distributions for the shuffled amino acid sequences, while the dashed lines show simple exponentials 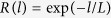.

Figure 7 shows that broad interval distributions can be observed exclusively in the adhesion and invasion factors located in the membranes of pathogenic *Firmicutes*, that have a single cell membrane and a thick cell wall (see Fig. 7(a)), in contrast to *Proteobacteria* having two membranes and a thin cell wall (see Fig. 7(b)). Notably, cytosine (C) is characterized by a heavy tail distribution in proteins from both *Firmicutes* and *Proteobacteria* that might be a reflection of its role in the disulfide bond formation that stabilizes the protein tertiary structure.

In contrast to the adhesion factors, associated with membrane, no heavy tail distributions of amino acids were found in the DNA-binding regulatory proteins (transcription factors), which are located in cytosol and obey similar functions in both bacterial groups (Fig. 7(c,d)). Again, simple exponential distributions can be observed for shuffled amino acid sequences in all studied protein groups. These facts confirm that considerable discrepancies in the interval distributions between amino acid residues can be observed rather for proteins with significant structural differences like presumable *α*-helixes or *β*-sheets in their secondary structure, localization in the cell membrane, outside or inside the cell that are explicitly reflected in the amino acid residues arrangement.

Next using the structural differences of the membrane-associated adhesion and invasion factors of *Firmicutes* and *Proteobacteria* as a prominent example of a structure-driven protein property, we check whether they can be classified using the mass spectra of proteolytic cleavage fragments following a similar algorithm as for the secondary structure.

#### Mass spectrometry simulation

**Figure 8:**
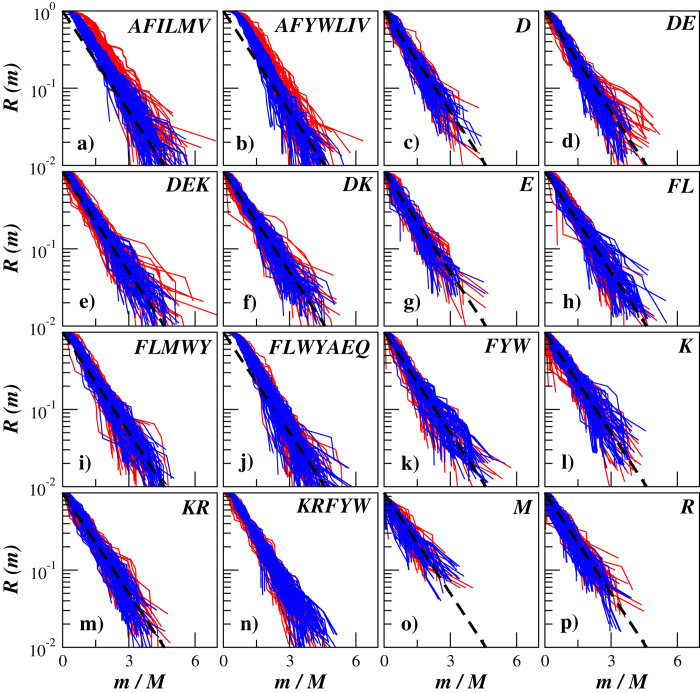
The exceedance probability distributions of oligopeptide masses in the adhesion and invasion factors of pathogenic *Firmicutes* (red) and *Proteobacteria* (blue) exemplified for 100 randomly selected proteins from each group. For comparison, the dashed lines show simple exponentials 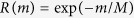.

Figure 8 shows the exceedance probability distributions of oligopeptide masses in adhesion and invasion factors of pathogenic *Firmicutes* and *Proteobacteria* exemplified for the same 16 different cleavage rules as used previously for the prediction of protein secondary structure. The figure shows that there are moderate discrepancies between the oligopeptide mass distributions, while they are less pronounced compared to the proteins with different secondary structure.

#### Statistical analysis and classification

**Figure 9:**
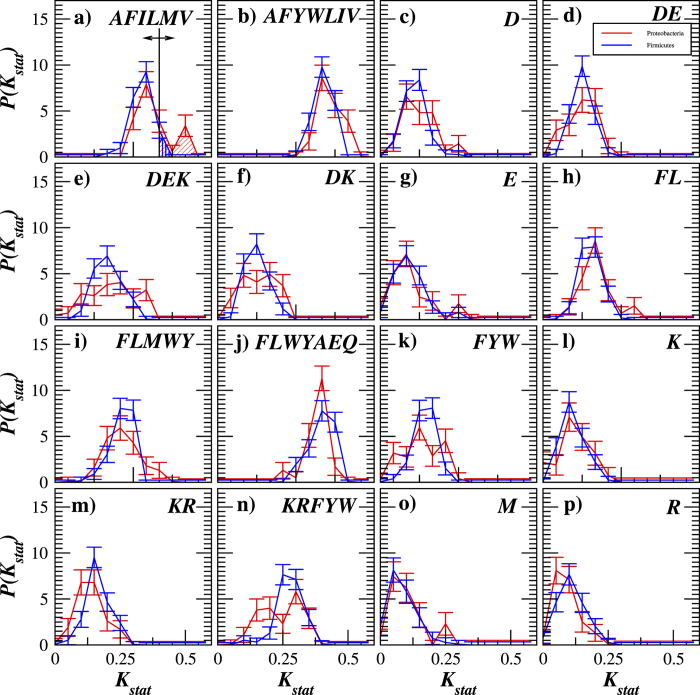
The empirical PDF estimates of *K*_stat_ values in the adhesion and invasion factors of pathogenic *Firmicutes* (red) and *Proteobacteria* (blue). The black vertical line in (**a**) indicates a variable decision threshold 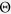 used to calculate a ROC-curve, while the diagonal and the vertical filled areas denote the hit rate and the false alarm rate for the chosen threshold 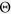, respectively.

**Figure 10:**
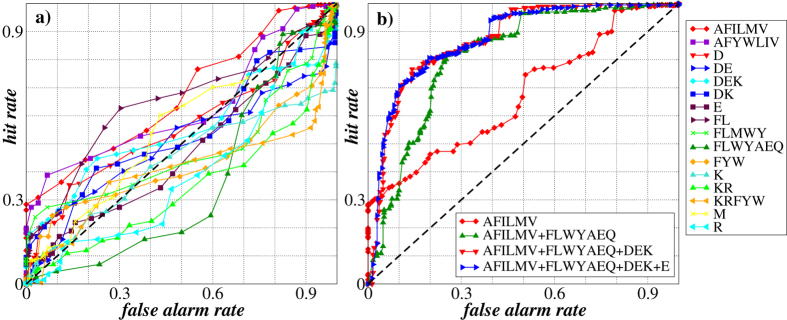
(**a**) The ROC curves characterizing the potential prediction accuracy of the adhesion and invasion factors of pathogenic *Firmicutes* and *Proteobacteria* according to the oligopeptide mass distributions exemplified for 16 different cleavage rules. (**b**) The ROC curves for the binary logistic regression models representing one to four best linear combinations of predictors using different cleavage rules. The areas under the curves (AUC) are 0.673, 0.813, 0.871 and 0.873, while the Nagelkerke 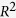 coefficients of determination are 0.164, 0.425, 0.516 and 0.561 for one, two, three and four cleavages used, respectively.

Figure 9 shows the 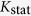 value distributions for 16 cleavage rules of adhesion and invasion factors of pathogenic *Firmicutes* and *Proteobacteria*. Like in the previous example (Fig. 6), we compare 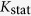 against a variable threshold 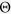 and plot ROC curves for each cleavage rule (shown in Fig. 10(a)). The figure shows that many ROC curves are fluctuating around the diagonal with exceptions for several cleavage rules, again including Thermolysin (AFILMV) and Pepsin with 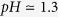 (FL) as well as Pepsin with 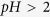 (FLWYAEQ). While the classification accuracy for any single cleavage rule remains below 70% that is hardly acceptable, combining two or three cleavage rules leading to the increase of AUC up to 0.81 and 0.87, respectively, in this case provides a considerable improvement.

### Validation of the prediction accuracy for various structural, localization and functional properties of proteins

Next we test the potential power of the fragment mass distribution analysis to predict a wide range of structural, localization and functional properties in different protein groups. For this large-scale validation analysis we employ data from the general proteomic databases UniProtKB/Swiss-Prot^41^ and RSCB PDB^42^, from the databases of intrinsically disordered proteins DisProt^43^ and Ideal^44^ as well as from the transmembrane protein database PDBTM^45^ and the bacterial virulence factors database VFPB^46^. We follow a unified procedure to prepare the datasets for the analysis including removal of items containing illegal symbols and elimination of redundant (duplicate) entries wherever applicable. We also do not consider short proteins containing less than 100 amino acid residues, since their interval and mass distributions exhibit pronounced finite size effects, especially for those cleavage rules where only one or two amino acid residues specify cleavage sites. Next for each sequence we simulate all 16 cleavages, estimate their (normalized) mass distributions and calculate 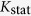 values that characterize their deviation from the theoretical (null hypothesis) exponential distribution. For predicting binary attributes, to combine 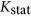 values obtained for different cleavages, we use binary logistic regression models. For predicting non-binary features like protein structural classes or functional groups, we perform a standard discriminant analysis procedure. To facilitate cross-validation, for all studied protein groups we first randomly select about 70% of proteins and use them to fit the prediction model parameters. Next, we test the model against both the (70%) selected proteins and the remaining (30%) unselected proteins. All large-scale statistical analysis for this subsection has been performed using SPSS Statistics software. Particular options used in the prediction model design and its statistical validation are outlined in the Methods section. The validation results including the model parameters and the prediction accuracies are summarized in Supplementary information available online.

#### Predicting structural properties of proteins

First we focus on the structural properties of proteins. As a very general and nonspecific testbed, we choose low-homology datasets known as 1189 and 25pdb that include globular proteins belonging to all four SCOP structural classes. These datasets has been widely used to test the efficacy of protein structural class predictors based on sequence analysis^18^,^29^,^30^,^31^. Our results (summarized in Supplementary Tables S1.1.1 and S1.1.2) indicate that the predictability of SCOP structural classes from mass distribution data barely exceeds 40% accuracy that is well below the performance of the sequence analysis based methods. Moreover, even to reach this accuracy, the prediction model requires data from 10 out of 16 tested cleavages that appears very impractical. Thus at this point we conclude that prediction of SCOP structural classes of globular proteins by mass distribution analysis remains challenging and alternative methods are currently preferable.

Next we repeat similar analysis after adding a fifth group containing intrinsically disordered proteins obtained from either DisProt^43^ or IDEAL^44^ databases to the existing datasets. Remarkably, intrinsically disordered proteins could be more easily distinguished from the SCOP-classified protein groups, showing above 60% accuracy (see Supplementary Tables S1.2.1–S1.2.5). When reducing to a binary criteria, i.e., distinguishing between all SCOP-classified and all intrinsically disordered proteins, the accuracy reached 65–70% for a single cleavage and about 75% for an optimal combination of 3–4 cleavages (see Supplementary Tables S1.2.3 and S1.2.6).

Further we focus on a more specific example that we already used above to illustrate our approach, namely the classification of transmembrane proteins between those carrying *α*-helixes and *β*-sheets in their secondary structure. Since the total number of proteins in the *α*-helical group is more than 10 times higher than in the *β*-barrel group, for the validation tests we compare the *β*-barrel group against randomly selected subsets of the *α*-helical group. For 10 different subsets, the overall prediction accuracy remained between 85 and 93%. Results for a single representative test sample are shown in Supplementary Tables S1.3. The tests have confirmed that using a single cleavage by Thermolysin is sufficient to obtain nearly 90% accuracy, while including further cleavages into the model did not lead to significant improvements in the predictability, in agreement with our above findings by ROC analysis.

#### Predicting cellular localization and localization-associated properties of proteins

Here we again started with tests on low homology datasets obtained from the UniProtKB/Swiss-Prot database^41^. First, we test whether our analysis can help distinguishing between soluble (localized either in the cytosol or in the cell nucleus) vs membrane localized proteins. Large-scale analysis of more than 200000 proteins provided promising 76.5% accuracy for a single cleavage by GluC(phosphate)+LysC (denoted as DEK) and nearly 80% accuracy when combining with three other cleavages. Remarkably, all amino acids involved in the best DEK cleavage, belong to the external hydropathy group, the property that has been recently and successfully used in sequence-based protein structural class predictions methods^31^. These accuracies are also in a nearly perfect agreement for the selected and unselected test groups (see Supplementary Tables S2.1.1).

Next we focus on the analysis of membrane associated proteins and try to distinguish between monotopic vs bi- and polytopic proteins. Once more the same cleavage by GluC(phosphate)+LysC shows the best accuracy of 71% that can be further enhanced to nearly 80% by adding three other cleavages (see Supplementary Tables 2.1.2). Finding single- and multi-pass structures in transmembrane proteins is possible with nearly 75% accuracy now with the help of cleavage by Chymotrypsin, while adding three more cleavages including recently used GluC(phosphate)+LysC as well as widely spread Trypsin leads to approximately 80% prediction accuracy (see Supplementary Tables 2.1.3).

Once more considerably lower performance has been observed in tests for non-membrane proteins. In particular, for distinguishing between proteins localized either in the cytosol or in the cell nucleus, best single-cleavage accuracy of 70% is obtained for Proteinase K, and adding further cleavages barely enhances the predictability (see Supplementary Tables S2.1.4).

When focusing on high homology data from pathogenic *Firmicutes* and *Proteobacteria* (obtained from^46^) and distinguishing between membrane-associated and DNA-binding (cytosolic) proteins either by localization or by host bacteria phylum, 65–75% single-cleavage based accuracy has been obtained that could be further enhanced up to 80–90% by adding 2–3 extra cleavages (see Supplementary Tables S2.2.1 to 2.2.3).

#### Predicting functional properties of proteins

Finally, we look at the functional properties of proteins and check whether the post-cleavage mass distribution analysis can contribute to the classification of proteins as belonging to a certain functional group. Among non-membrane proteins obtained from the UniProt/Swissprot database^41^, we first focus on three large groups, namely cytoskeletons, enzymes and transcription factors. Despite of using all 16 cleavages, the classification accuracy barely exceeded 53% that is well below acceptable level (see Supplementary Tables S3.1.1). When reducing to a binary case and trying to predict only cytoskeletons among other non-membrane proteins, approximately 60% accuracy could be obtained with popular Trypsin cleavage, while further improvements were much less significant (see Supplementary Tables S3.1.2). Slightly higher (about 65%) accuracy could be obtained for the distinction between enzymes and transcription factors (see Supplementary Tables S3.1.3).

Again much better prediction efficacy could be obtained for the *α*-helical membrane proteins from the PDB database^42^. Using discriminant analysis more than 70% of proteins could be correctly attributed to one out of five functional groups including ATP-binding proteins, ABC-transporter channels, G-protein receptors, photosynthetic proteins and bacterial rhodopsines (see Supplementary Tables S3.2). We also tested a couple of binary classification cases, that are specific to certain organisms, for example for *Bacilli* which do not exhibit photosynthesis. While distinguishing between ATP-binding and transporter proteins required three cleavages to reach nearly 80% accuracy, another binary classification between transporters and G-protein receptors required only a single cleavage by Trypsin/Chymotrypsin that alone led to more than 75% accuracy (see Supplementary Tables S3.3 and S3.4, respectively). Interestingly, in this case other cleavages provided insignificant classification patterns and thus were not included in the analysis.

### Test examples

To emphasize the simplicity of our analysis, we implemented two simple test examples as spreadsheets (available as Supplementary datasets 1 and 2). In these examples, two *B.subtilis* proteins, the transmembrane ammonium transporter channel (AmtB) and the cytosol located glutamine synthetase (GS), are being consecutively tested against several binary classifiers. Each example contains multiple tabs, where the first tab summarizes the classifiers and illustrates the binary classification procedures based on logistic regression models, while the other tabs illustrate the calculation of 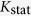 values for each simulated cleavage. In each tab except the first one, column B contains a sorted (in descending order) list of post-cleavage fragment masses *m*. In the test examples, these data has been obtained directly from the Expasy web service^49^,^50^,^51^ using a text format export option. In column C the masses from column B are being divided by the average fragment mass *M* to obtain the normalized mass spectrum. In columns D and E, the observational and the theoretical null hypothesis (exponential) exceedance probability distributions are being calculated, respectively. For the graphical representation of the distributions, one can plot columns D and E as a function of column C, respectively. Finally, column F is simply the difference between columns D and E. By definition, the maximum difference is the decision statistics used in the Kolmogorov statistical test, that we denote here as 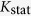.

Now let us turn to the first tab. For both AmtB and GS proteins (see Supplementary datasets 1 and 2), the first test is about their localization. Cleavage rules included in the prediction model, particular logistic regression equations as well as the decision threshold values are taken from Supplementary Tables S2.1.1. First, the best (according to validation results) single cleavage rule is tested, in this case GluC(phosphate)+LysC (denoted as DEK). Logistic regression coefficients 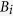 from the Supplementary Tables S2.1.1 (online) are inserted into the logistic regression equation 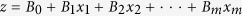 to calculate *z* statistics that is next used to calculate 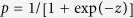. Finally, *p* is compared against the decision threshold 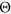, also obtained from Supplementary Tables S2.1.1. According to the given criteria, the prediction is made. Since the prediction accuracy usually increases with increasing the number of cleavages used in the model, we confirm our prediction in a second step, that now includes data from two cleavages (DEK+FLMWY). For the two-compound model, *p* deviates even further from the threshold 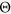, indicating that incorporating the second cleavage into the model additionally supports the initial prediction.

In the following, a similar procedure is repeated for a number of binary classification criteria. Since in the first test the AmtB protein was (correctly) predicted as a membrane-associated protein, in the next step we test whether it is a monotopic or a transmembrane protein. After we have confirmed it is a transmembrane protein, in tests 3 and 4 we try to predict its organization in the membrane (single- or multi-pass) as well as its secondary structure (*α*-helical or *β*-barrel). In the fifth test, we find that its host organism likely belongs to *Firmicutes*. Finally we look for the most probable functional role of this protein in the living cell. Since most *Firmicutes* do not exhibit photosynthesis, some functional groups such as photosynthetic proteins and rhodopsines are not being considered. Furthermore, since our predictions indicate that it is a transmembrane protein, we also do not consider the ATP-binding functional group that contains only monotopic proteins. Thus among large functional groups only transporters and G-protein receptors remain in the analysis that allows us again to reduce to a binary criteria. Finally, we have predicted that the AmtB protein is likely a transporter protein.

A similar design is used in the GS example (see Supplementary dataset 2). Since in the very first test is it is classified as a cytosolic protein, we next test whether in could belong to the cytoskeleton group and finally in the third test arrive at the decision that it is most likely an enzyme.

Using the above test examples, we additionally studied the robustness of our approach against missing cleavages. For the AmtB example, we performed similar analysis allowing one to five consecutive missing cleavages, using an embedded feature of the Expasy web service^49^. The results are available as Supplementary datasets 3, 4, 5, 6 and 7, corresponding to one, two, three, four and five consecutive missing cleavages, respectively. In these Supplementary datasets, correct predictions are highlighted by green, while false predictions are highlighted by red and ambiguous cases by yellow. The spreadsheet structure is identical to that one in the original AmtB example (available as Supplementary dataset 1). Our resutls indicate that single missing cleavages do not affect the prediction results (see Supplementary dataset 3), while allowing for two consecutive missing cleavages disrupts the prediction of the multi-pass structure of the AmtB transmembrane protein (see Supplementary dataset 4). Similar consequences can be drawn for three and four consecutive missing cleavages (see Supplementary datasets 5 and 6). Remarkably, since other considered predictors are irrelevant to either single- or multi-pass structure, they still perform accurately with up to four missing cleavages allowed. Finally, with five consecutive missing cleavages allowed, localization and host organism predictors start providing ambiguous results. In particular, the localization of AmtB in the cellular membrane is correctly predicted by using the most informative cleavage, while incorporating further data from other cleavages into the model leads to the inversion of the original prediction. Additionally, the host bacteria phylum is wrongly predicted at a first step, while adding further cleavages inverse the prediction to the correct one (see Supplementary dataset 7).

In contrast, for the GS example, a similar test revealed that its functional group prediction fails even under single missing cleavage allowance (see Supplementary dataset 8). When two consecutive missing cleavages are allowed, only the prediction of cellular localization remains possible. While failing at the initial step when a single cleavage is involved, after adding data for other cleavages it finally leads to a correct classification (see Supplementary dataset 9). Other predictors lead to either false or ambiguous results, indicating that the robustness of our approach is considerably lower for cytosolic proteins than for transmembrane proteins. We attribute this fact to the characteristic structure-associated non-random patchiness in the arrangement of monomers in the primary polypeptide chain of transmembrane proteins leading to pronounced deviations of their inter-amino-acid interval and post-cleavage mass distributions from the theoretical (null hypothesis) exponential functional form. In contrast, for soluble proteins the deviations from this null hypothesis assumption appear much less pronounced, leading to the reduction in the overall prediction accuracy and especially in the decrease of robustness against missing cleavages. We think that this is the major conceptual limitation of our approach, that it requires at least one of the analyzed protein groups to exhibit characteristic structural features clearly reflected in the “patchy” arrangement of monomers in their primary polypeptide chain, with transmembrane proteins being a prominent example.

Similar examples could be designed for many other proteins, while the exact sequence of tests will depend on the outcome of previous tests. Of course one has to take into account that prediction errors in one test will also disrupt the accuracy of the following tests, and the overall prediction accuracy will reduce drastically with the increasing number of consecutive tests affected by each other. However, in most realistic experimental designs isolated proteins usually do not appear “out of the blue”, and at least some information about its host organism, cellular localization or some other specific properties is commonly available to the investigator. Thus in most practical scenarios making either one or two or rarely three prediction tests is already a significant contribution to the understanding of the studied protein role in the living cell or even more often for ruling out unlikely candidate proteins to reduce the number of experiments and save laboratory costs.

We like to note that this is just a simple test design that does not aim to serve as an end user ready bioinformatic tool. Instead, it is suited only for demonstration of the potential applicability of the suggested approach. Contrast to this test tool that contains (fairly unrealistic) equal probability assumptions for all other parameters except the mass distribution discrepancy, an end user ready prediction tool should incorporate many other (already known) issues such as different fractions of amino acids in primary polypeptide chains of different proteins, existing combinations of protein structural, localization and functional properties and so on. Presented test model design takes neither of those factors into account. For the same reason, we did not provide similar test examples for multiple classification criteria. The efficacy of multiple classification criteria design depends drastically on the appropriate choice of metrics, background probabilities as well as other factors mentioned above, that is beyond the scope of this paper. Finally, by eliminating other factors or considering most trivial equal probability hypothesis we could quantify explicitly the amount of information about a given protein that can be extracted using exclusively the suggested approach based on the analysis of the distributions of polypeptide cleavage fragment masses.

## Conclusion and Outlook

To summarize, we have suggested a statistical approach to predict several structure-associated properties of arbitrary proteins directly from the mass distributions of their proteolytic cleavage fragments without reconstruction of primary polypeptide chains. As we show below, in some cases this leads also to the successful prediction of cellular localization and functional group of the studied proteins. The suggested approach is especially efficient in those cases when the localization and function of studied proteins are largely associated with their structural conformations, which in turn are governed by the arrangement of amino acid residues in their primary sequence. In turn, the arrangement of amino acid residues is explicitly reflected in the functional forms of the mass distributions of the polypeptide chain fragments after its amino acid-specific proteolytic cleavage.

Our approach has been originally inspired by the recent studies of interval statistics in various complex systems including biological polymer structures^12^,^52^,^53^,^54^. Here we show that a number of structural, localization and functional properties of proteins can be successfully predicted with 60–80% and in some cases up to 90% accuracy from their cleavage fragment mass distributions assessed by well-established MALDI-TOF technique.

In contrast to the existing methods to predict structure and function of the analyzed protein from its primary amino acid sequence that are largely based on similarity principles and thus require highly homologous proteins to be reviewed, our approach is based on the direct analysis of the mass distribution of polypeptide cleavage fragments that contains the distinct reflection of the protein structural conformation complexity. Therefore we think that the major advantage of our approach is that it neither requires nor reconstructs the protein sequence and thus can be used to characterize unknown proteins directly from their post-cleavage mass spectrometry data. Besides that, the overall statistical analysis procedure appears quite simple and easy to implement.

An obvious limitation of our approach is that it bases solely on the analysis of the arrangement of amino acid residues in the primary polypeptide chain by its comparison with the random (null hypothesis) assumption. Thus it requires that at least one of the analyzed protein groups exhibits some characteristic structural features clearly reflected in the arrangement of monomers in their primary polypeptide chain. For that reason, our approach distinguishes well between proteins with different structural patterns like transmembrane proteins, while exhibits considerably lower accuracy for distinguishing proteins without pronounced structural discrepancy like globular proteins with different functions.

Our first results indicate that for the transmembrane proteins the secondary structural class (either *α*-helical or *β*-barrel) can be correctly predicted in approximately 90% cases by using a single cleavage by Thermolysin, while three best cleavage rules are required to correctly predict a membrane-associated protein as belonging to either *Firmicutes* or *Proteobacteria* in about 80% cases. Results of further extensive statistical validation of the suggested approach on several hundreds of thousands of reviewed proteins indicate that the cellular localization of proteins (either soluble of membrane-associated, monotopic or transmembrane) can be predicted with 70–80% accuracy using the mass-spectrometry data after a single cleavage by GluC(phosphate)+LysC. While the correct attribution of protein to one of four SCOP structural classes is low (barely exceeding 40% accuracy), we could distinguish about 3 out of 4 intrinsically disordered proteins from SCOP-classified proteins. Moreover, using binary classification matrices, we could identify the putative protein function with about 60–80% accuracy.

We like to note that the above accuracy values are obtained by the analysis of mass distribution discrepancies only. To our opinion, a substantial improvement could be achieved by combining our approach with known parameters such as different fractions of amino acids in the primary polypeptide chains of different proteins, existing combinations of protein structural, localization and functional properties as well as other parameters that are commonly used in protein prediction tools. As an outlook, we think that another perspective way to improve the prediction accuracy is a proper selection of the datasets used to learn the statistical models. Recent evidence indicates that appropriate selection of proteotypic peptides can considerably improve the accuracy of various statistical models used in proteomics^55^.

The proposed methodology, to our opinion, could deserve a number of practical applications. It appears especially useful when the primary sequence of the protein is not available (gene is not sequenced) and thus protein characteristics cannot be predicted by sequence alignment methods. Therefore, distributions of inter-amino-acid intervals or polypeptide cleavage fragment masses could be used as supplemental information in various protein structure prediction algorithms. Moreover, in many cases preliminary estimation of structural properties, cellular localization and functional group of the analyzed protein could significantly reduce the number of candidates for further analysis. As an outlook, similar statistical principles could be of interest to improve the algorithms of tandem mass spectrometry data analysis as well as *de novo* protein sequence reconstruction.

## Methods

### Interval distributions and their interpretation

Interval distributions between the positions of certain items in a sequence are important quantities that are widely used to characterize both structural and dynamical features of complex systems in physics, biology, geosciences, climate, finance and many other applications. In time series analysis interval distributions between the consecutive occurrences of certain events (e.g. when the series exceeds a certain value, fits within a given range, or crosses a certain threshold) explicitly reflect the persistence properties of the analyzed system. In a fully random (uncorrelated) data series the expected probability density function (PDF) of the intervals between such events follows a simple exponential 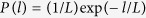, where *L* is the average interval. In linearly long-range correlated data, one expects the asymptotic PDF of the intervals to follow a stretched exponential 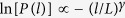, with exponent 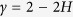
56,57,58, where *H* is the Hurst exponent characterizing the LRC. In the presence of nonlinear correlations, the PDF gets even broader and decays asymptotically by a power-law 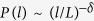, where the exponent *δ* decreases when the LRC gets more pronounced^52^,^53^,^54^.

To understand the reflection of the protein structure and amino acid arrangement in the interval distributions, let us follow the polypeptide chain in one direction (e.g. from N-term to C-term) and mark all positions where a particular amino acid residue (e.g. ‘R’) characterized by its specific mass value is observed (see Fig. 2(a)). First, the average interval between the marked positions would be inversely proportional to the frequency of the occurrence of ‘R’ in the polypeptide chain. Next, if the marked positions are allocated randomly along the polypeptide chain, then we expect the exponentially decaying distribution of the intervals between them. In contrast, if marked positions tend to cluster indicating some structural inhomogeneity, short intervals would follow short intervals, while long intervals would follow long intervals, this way leading to the distributions that are considerably broader than a simple exponential (see Fig. 2(b)). This situation corresponds to the presence of correlations in the sequence of numbers assigned to each amino acid (e.g. their molecular masses).

Commonly empirical PDF estimates are obtained from the histograms of observational data, the method that is hardly applicable to short protein sequences where often only a few occurrences of a given amino acid residue could be observed. Therefore instead of using 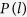 here we focus on the probability that the interval exceeds a given value 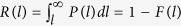, where 
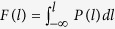
 is the cumulative distribution function (CDF). To characterize the arrangement of all 20 amino acid residues, similar analysis is repeated 20 times for each protein sequence providing a family of exceedance probability distributions 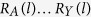. To compare the functional forms of the distributions for different amino acid residues that are observed more or less frequently in the polypeptide chain, they are presented in scaled form 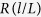, where *L* is the average interval between particular amino acid residues in a given polypeptide chain.

### Mass spectrometry and mass distributions

**Figure 11:**
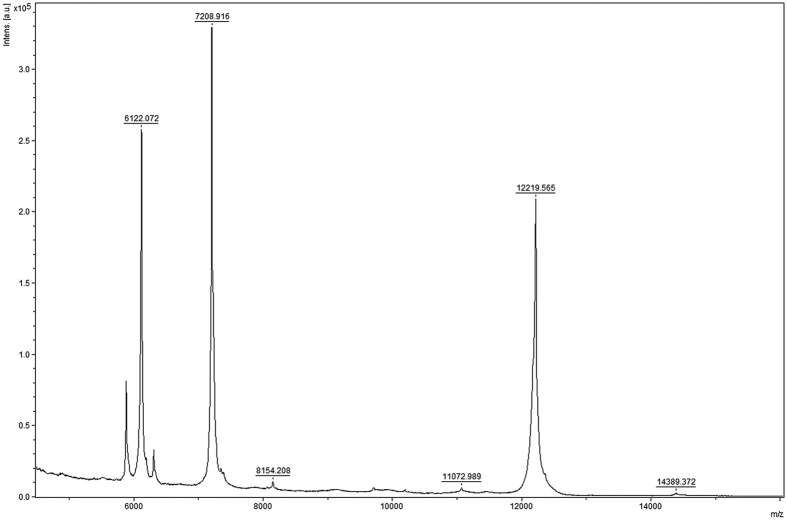
An example of an experimental mass spectrum of a protein after proteolytic cleavage. Ribonuclease binase from *B.pumilus* was cleaved by trypsin and the cleavage fragments were analysed on HPLC LC-MS/MS system (Bruker, Germany)^60^. The numbers denote determined oligopeptide masses.

Figure 11 shows a representative example of an experimental MALDI TOF mass spectrum of polypeptide chain fragments of ribonuclease protein from *B.pumilus* after its cleavage by trypsin (cleavages at ‘K’ and ‘R’). Typical experimental mass spectra contains a lot of background “chemical noise”^59^ as well as artifacts due to unsuccessful or unspecific cleavages at some sites. When the cleavage is performed at multiple sites, for example by Thermolysin (AFILMV), Proteinase K (AFYWLIV) or Pepsin with 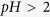 (FLWYAEQ), the “signal-to-noise” ratio in the mass spectrum decreases. Therefore the interpretation of the mass spectra is typically limited to the detection of peaks above the background noise. Since the molecular masses of all amino acid residues are known with high accuracy, only those peaks that correspond to a possible combination of amino acid residues masses and thus can be associated with a realistic oligopeptide can be selected. Next for this “discrete” mass spectrum the distribution of oligopeptide masses can be obtained. The shapes of the oligopeptide mass distributions are mainly determined by their size distributions that are directly defined by the interval distributions between those amino acid residues that specify cleavage positions. Thus a broader interval distribution leads to a broader mass distribution, and vice versa. The relative fraction of different amino acid residues in a given protein provides rather secondary effect on the shape of the (normalized) distribution that is also hard to account when the protein sequence is not available.

The simulation of the proteolytic cleavage of the proteins was performed for 16 different cleavage rules corresponding to the commercially available enzymes/reagents^47^ summarized in Table 1. Like for the intervals, the normalized exceedance probability distribution 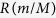 was calculated, where *M* is the average oligopeptide mass for the particular protein and a given cleavage rule. To quantify the deviation of the oligopeptide mass distributions from the theoretical (null hypothesis) exponential distribution, we employ the standard Kolmogorov statistics 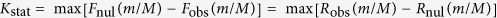. To find useful predictors we next calculate 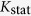 values for each considered protein and each cleavage rule. To distinguish between two protein groups, those cleavage rules where the distributions of 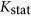 for proteins from different groups have the minimum overlap, appear the most powerful predictors.

### ROC analysis

Next for each cleavage rule we suggest a decision threshold 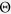. If 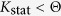, we predict that the protein belongs to the first group. If 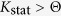, we predict that the protein belongs to the second group. For an explicit quantification of the prediction accuracy, for any given threshold value 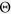 one can calculate the fraction of correctly predicted proteins from the second group also known as the hit rate as well as the fraction of proteins from the first groups falsely predicted as belonging to the second group known as the false alarm rate (exemplified by filled areas under the curves in Fig. 5(a)). By varying the decision threshold 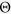, one can optimize the predictor performance according to a given criteria such as maximum overall accuracy, maximum tolerable false alarm rate and so on. For an overall quantification of the predictive power, one can plot the hit rate as a function of the false alarm rate for all possible threshold values 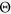 this way obtaining the so-called receiver operator characteristic (ROC) curve (exemplified in Figs 6 and 10)^40^.

At 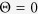, both the hit rate and the false alarm rate equals one, and the ROC curve starts from the upper right corner. If 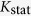 distributions in both groups would be identical, the probability of exceeding any threshold value 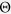 by them will be always equal, and thus the ROC-curve would follow a diagonal (shown by dashed line in Figs 6 and 10). In contrast, if the respective distributions would have no overlap (e.g. the largest 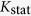 value in the first group will be below the smallest 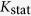 value in the second group), the ROC curve would follow first the horizontal and then the vertical axis, forming a rectangular step. In most realistic scenarios, the distributions partially overlap, and the ROC curve is below the diagonal when the fragment mass distributions are broader for the first protein group, and is above the diagonal when the fragment mass distributions are broader for the second protein group. For any given decision threshold 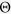, the ROC curve provides explicitly the fraction of correctly classified proteins from each group as well as the fraction of misclassification cases. The overall predictor efficacy is given by the area under the curve (AUC), if the ROC curve is above the diagonal, or below the curve, if the ROC curve is below the diagonal. For the perfect prediction, AUC = 1, while for no predictive power (like random guess) AUC = 0.5.

### Logistic regression model

To test whether the prediction accuracy can be improved by using the combination of cleavage rules, we followed a stepwise procedure outlined below. In the first step, for each protein we calculated 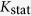 values for all considered 16 cleavage rules according to^48^ that we consider as potential predictors. In the second step, we eliminated those cleavage rules that did not provide statistically significant differences between the predictor values in the studied protein groups according to the Mann-Whitney criterion. In the third step, possible combinations of the remaining predictors are stepwisely included into the logistic regression model. Particular order of the predictors to be included in the model is determined by their Wald statistics that is proportional to their significance levels as single classifiers. Once an additional classifier is included, one calculates the probability that the studied protein belongs to one of the considered groups 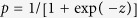, where 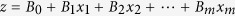 is a linear combination of considered predictor values, given that 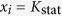 for the *i*th cleavage rule. In the fourth step, the performance of the resulting model is tested by calculating *p* for each protein and comparing it against a varying decision threshold 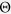. Whether *p* is smaller or larger than 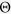, the protein is classified as belonging to the first or the second group, respectively. By comparing our results against the (known) classification of the proteins for each 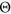, we next obtained the ROC curve, now always above the diagonal, since the appropriate signs for each predictor are accounted in the equation for *z* (see Figs 6(b) and 10(b)). Inclusion of additional predictors into the combined model was stopped once no significant enhancement of the ROC curve and thus no further increase in the prediction accuracy could be observed. Additionally we calculated the Nagelkerke 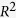 coefficient of determination that provides the fitting quality of the logistic regression model for the analyzed protein group (denoted in the captions of Figs 5(b) and 9(b)).

### Discriminant analysis

To test the potential predictability of the protein properties according to various non-binary classifications, we employed the disriminant analysis procedure. Like in the binary classification case, in the first step for each protein we calculated 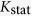 values for all considered 16 cleavage rules according to^47^ that we consider as potential predictors. Next, single predictors are included into the combined model in a stepwise manner. At each iteration, the predictor that maximized the Mahalanobis distance between the two closest groups was included into the combined model. Inclusion of additional predictors into the combined model was stopped once no significant increase in the prediction accuracy could be observed.

## Additional Information

**How to cite this article**: Bogachev, M. I. *et al.* Statistical prediction of protein structural, localization and functional properties by the analysis of its fragment mass distributions after proteolytic cleavage. *Sci. Rep.* **6**, 22286; doi: 10.1038/srep22286 (2016).

## Supplementary Material

Supplementary Information

Supplementary Dataset 1

Supplementary Dataset 2

Supplementary Dataset 3

Supplementary Dataset 4

Supplementary Dataset 5

Supplementary Dataset 6

Supplementary Dataset 7

Supplementary Dataset 8

Supplementary Dataset 9

## Figures and Tables

**Figure 1:**
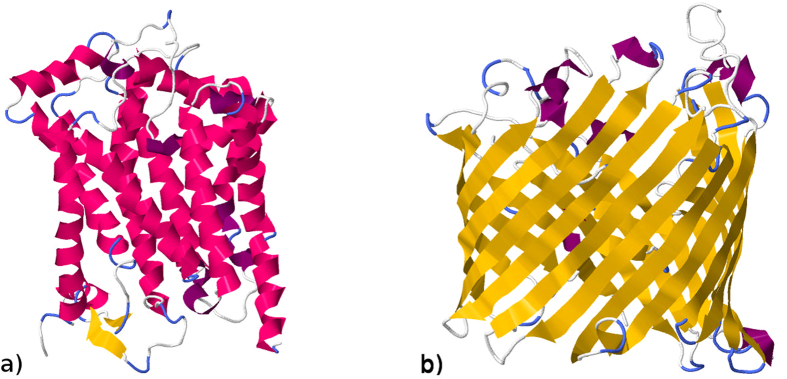
3d reconstructions of two transmembrane proteins, (**a**) cytochrome B from *Bos taurus* and (**b**) sucrose porin from *Salmonella enterica* carrying *α*-helixes and *β*-sheets in their secondary structure, respectively. The structures were predicted using the Phyre2 molecular modeling algorithm^20^.

**Table 1:** The summary of cleavage rules considered in this study.

Short notation	Enzyme/Reagent	Cleavage position(s)	Exceptions from the cleavage rule
AFILMV	Thermolysin	N-terminal side of A, F, I, L, M, V	if D or E is N-term to A, F, I, L, M, V
AFYWLIV	Proteinase K	C-terminal side of A, F, Y, W, L, I, V	—
D	Microwave-assisted formic acid hydrolysis	C-terminal side of D	—
DE	Glu C (phosphate)	C-terminal side of D or E	if P is C-term to D or E, or if E is C-term to D or E
DEK	Glu C (phosphate)+Lys C	C-terminal side of D, E and K	if P is C-term to D or E, or if E is C-term to D or E
DK	Asp N/Lys C	N-terminal side of D, C-terminal side of K	—
E	Glu C (bicarbonate)	C-terminal side of E	if P is C-term to E, or if E is C-term to E
FL	Pepsin 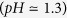	C-terminal side of F, L	—
FLMWY	Chymotrypsin (F/L/M/W/Y)	C-terminal side of F, L, M, W, Y	if P is C-term to F, L, M, W, Y, if P is N-term to Y
FLWYAEQ	Pepsin (*pH* > 2)	C-terminal side of F, L, W, Y, A, E, Q	—
FYW	Chymotrypsin (F/Y/W)	C-terminal side of F, Y, W	if P is C-term to F, Y, W, if P is N-term to Y
K	Lys C	C-terminal side of K	—
KR	Trypsin	C-terminal side of K or R	if P is C-term to K or R
KRFYW	Trypsin/Chymotrypsin	C-terminal side of K, R, F, Y, W	—
M	CNBr	C-terminal side of M	—
R	Arg C	C-terminal side of R	if P is C-term to R
