# Supplementary material for: Statistical prediction of protein structural, localization and functional properties by the analysis of its fragment mass distributions after proteolytic cleavage
Source: Sci Rep. 2016 Feb 29;6:22286. doi: 10.1038/srep22286 (PMC4770285; doi:10.1038/srep22286)
Supplement: Supplementary Information [file srep22286-s1.pdf]

Supplementary information

to «Statistical prediction of protein structural, localization and functional properties by the analysis of its fragment mass distributions after proteolytic cleavage»
by Mikhail Bogachev, Airat Kayumov, Oleg Markelov and Armin Bunde

Prediction models and validation of their accuracy for various protein groups

Table of Contents

S1 Classification according to structural properties 2
S1.1 Classification by SCOP (Structural Classification Of Proteins) 2
S1.1.1 25pdb low homology dataset 2
S1.1.2 1189 low homology dataset 4
S1.2 Comparison of SCOP-classified proteins vs Intrinsically disordered proteins 6
S1.2.1 25pdb low homology dataset + intrinsically disordered proteins (obtained from the *DisProt* database) 6
S1.2.2 25pdb low homology dataset + intrinsically disordered proteins (obtained from the *Ideal* database) 8
S1.2.3 25pdb low homology dataset + intrinsically disordered proteins (obtained from the *Ideal* database) 10
S1.2.4 1189 low homology dataset + intrinsically disordered proteins (obtained from the *DisProt* database) 11
S1.2.5 1189 low homology dataset + intrinsically disordered proteins (obtained from the *Ideal* database) 13
S1.2.6 1189 low homology dataset (subset) + intrinsically disordered proteins (obtained from the *Ideal* database) 15
S1.3 Classification of transmembrane proteins (subset obtained from http://pdbtm.enzim.hu) 16
S2 Classification by localization properties 17
S2.1 Low-homology data obtained from UniProtKB/Swiss-Prot database 17
S2.1.1 Prediction of membrane associated proteins 17
S2.1.2 Classification of membrane associated proteins 18
S2.1.3 Classification of polytopic transmembrane proteins 20
S2.1.4 Classification of non-membrane proteins 21
S2.2 High homology data from pathogenic *Firmicutes* and *Proteobacteria* (obtained from http://www.mgc.ac.cn/VFs/main.htm) 23
S2.2.1 Prediction of membrane proteins 23
S2.2.2 Prediction of membrane proteins (obtained from http://www.mgc.ac.cn/VFs/main.htm) 24
Supplementary S2.2.3 Prediction of membrane proteins by host organism 26
S3 Classification by functional properties 27
S3.1 Classification of non-membrane proteins obtained from UniProtKB/Swiss-Prot database 27
S3.1.1 General functional classification 27
S3.1.2 Prediction of cytoskeleton proteins among non-membrane proteins (Selected cases) 29
S3.1.3 Prediction of cytosolic non-cytoskeletal proteins by function 31
S3.2 Classification of membrane proteins obtained from RCSB PDB database 33
S3.3 Classification of membrane proteins (Selected example) 35
S3.4 Classification of membrane proteins (Selected example for transmembrane proteins of *Firmicutes* except *Heliobacteria,* no photosynthetic, no ATP-binding) 36

S1 Classification according to structural properties

S1.1 Classification by SCOP (Structural Classification Of Proteins)

S1.1.1 25pdb low homology dataset

Considered protein groups: all-alpha (1) vs all-beta (2) vs alpha/beta (3) vs alpha+beta (4)

Supplementary Table S1.1.1a
Variables Entered/Removed[a,b,c,d]

Step	Entered	Statistic	Between Groups
1	FLWYAEQ	.029	1 and 3
2	DEK	.071	1 and 4
3	FLMWY	.145	3 and 4
4	FYW	.189	2 and 4
5	KR	.211	2 and 4
6	K	.222	2 and 4
7	D	.225	2 and 4
8	AFYWLIV	.225	2 and 4
9	E	.226	2 and 4
10	AFILMV	.226	2 and 4

At each step. the variable that maximizes the Mahalanobis distance between the two closest groups is entered.

a. Maximum number of steps is 32.

b. Maximum significance of F to enter is .05.

c. Minimum significance of F to remove is .10.

d. F level. tolerance. or VIN insufficient for further computation.

Supplementary Table S1.1.1b
Standardized Canonical Discriminant Function Coefficients

	Function		
	1	2	3
AFILMV	.316	.273	-.395
AFYWLIV	.292	-.336	.238
D	-.371	.197	.161
DEK	.243	.291	.341
E	-.203	.307	-.096
FLMWY	.122	.681	-.400
FLWYAEQ	.396	.069	.377
FYW	-.169	.052	.704
K	-.283	-.021	-.203
KR	.046	.308	.221

Supplementary Table S1.1.1c
Classification Results[a.b]

				Predicted Group Membership				
			Group	1	2	3	4	Total
Selected cases	Original	Count	1	74	18	36	26	154
			2	43	85	59	46	233
			3	35	38	130	31	234
			4	39	58	57	42	196
		%	1	48.1	11.7	23.4	16.9	100.0
			2	18.5	36.5	25.3	19.7	100.0
			3	15.0	16.2	55.6	13.2	100.0
			4	19.9	29.6	29.1	21.4	100.0
Unselected cases	Original	Count	1	24	11	19	11	65
			2	14	33	19	25	91
			3	15	18	49	5	87
			4	23	26	22	24	95
		%	1	36.9	16.9	29.2	16.9	100.0
			2	15.4	36.3	20.9	27.5	100.0
			3	17.2	20.7	56.3	5.7	100.0
			4	24.2	27.4	23.2	25.3	100.0

a. 40.5% of Selected original grouped cases correctly classified.

b. 38.5% of Unselected original grouped cases correctly classified.

S1.1.2 1189 low homology dataset

Considered protein groups: all-alpha (1) vs all-beta (2) vs alpha/beta (3) vs alpha+beta (4)

Supplementary Table S1.1.2a
Variables Entered/Removed[a.b.c.d]

	Entered	Removed	Statistic	Between Groups
1	DEK		.012	3 and 4
2	M		.033	3 and 4
3	AFILMV		.050	2 and 4
4	FLWYAEQ		.115	1 and 3
5	FYW		.149	2 and 4
6	E		.153	2 and 4
7	FLMWY		.156	2 and 4
8	D		.159	2 and 4
9		FYW	.145	2 and 4
10	KR		.182	2 and 4
11	FL		.183	2 and 4

At each step. the variable that maximizes the Mahalanobis distance between the two closest groups is entered.

a. Maximum number of steps is 32.

b. Maximum significance of F to enter is .05.

c. Minimum significance of F to remove is .10.

d. F level. tolerance. or VIN insufficient for further computation.

Supplementary Table S1.1.2b
Standardized Canonical Discriminant Function Coefficients

	Function		
	1	2	3
AFILMV	.529	-.359	-.341
D	-.080	.559	-.088
DEK	.447	-.111	-.019
E	.143	.413	.105
FL	.038	.502	-.224
FLMWY	.391	.084	-.415
FLWYAEQ	.359	.105	.654
KR	.220	.359	.480
M	.311	-.160	.126

Supplementary Table S1.1.2c
Classification Results[a,b]

			Group	Predicted Group Membership 1	2	3	4	Total
Selected cases	Original	Count	1	47	13	19	11	90
			2	30	78	25	18	151
			3	46	43	97	21	207
			4	30	42	32	20	124
		%	1	52.2	14.4	21.1	12.2	100.0
			2	19.9	51.7	16.6	11.9	100.0
			3	22.2	20.8	46.9	10.1	100.0
			4	24.2	33.9	25.8	16.1	100.0
Unselected cases	Original	Count	1	14	9	11	2	36
			2	13	27	10	8	58
			3	21	17	45	8	91
			4	10	13	15	5	43
		%	1	38.9	25.0	30.6	5.6	100.0
			2	22.4	46.6	17.2	13.8	100.0
			3	23.1	18.7	49.5	8.8	100.0
			4	23.3	30.2	34.9	11.6	100.0

a. 42.3% of Selected original grouped cases correctly classified.

b. 39.9% of Unselected original grouped cases correctly classified.

S1.2 Comparison of SCOP-classified proteins vs Intrinsically disordered proteins

S1.2.1 25pdb low homology dataset + intrinsically disordered proteins (obtained from the *DisProt* database)

Considered protein groups: all-alpha (1) vs all-beta (2) vs alpha/beta (3) vs alpha+beta (4) vs intrinsic_disprot (5)

Supplementary Table S1.2.1a
Variables Entered/Removed[a.b.c.d]

Step	Entered	Removed	Statistic	Between Groups
1	D		.005	2 and 4
2	DEK		.056	2 and 4
3	FLWYAEQ		.093	3 and 5
4	FYW		.120	1 and 4
5	KRFYW		.135	1 and 4
6	AFILMV		.147	2 and 4
7	K		.157	2 and 4
8	FLMWY		.163	2 and 4
9	KR		.169	2 and 4
10	AFYWLIV		.172	2 and 4
11	DE		.176	2 and 4
12	E		.177	2 and 4
13		DE	.175	2 and 4
14	FL		.176	2 and 4

At each step. the variable that maximizes the Mahalanobis distance between the two closest groups is entered.

a. Maximum number of steps is 32.

b. Maximum significance of F to enter is .05.

c. Minimum significance of F to remove is .10.

d. F level. tolerance. or VIN insufficient for further computation.

Supplementary Table S1.2.1b
Standardized Canonical Discriminant Function Coefficients

	Function			
	1	2	3	4
AFILMV	-.013	.398	-.170	-.490
AFYWLIV	.352	-.166	-.750	.122
D	.367	-.177	.161	.006
DEK	.069	.323	.008	.371
E	.265	.217	.045	.061
FL	.109	.062	.330	-.177
FLMWY	.313	.583	.102	-.062
FLWYAEQ	-.378	.272	.047	.464
FYW	.162	-.045	-.039	.537
K	.432	-.253	-.022	-.079
KR	-.334	.458	.415	-.244
KRFYW	.012	-.322	-.109	.464

Supplementary Table S1.2.1c
Classification Results[a.b]

			Group	Predicted Group Membership 1	2	3	4	5	Total
Selected cases	Original	Count	1	79	19	24	19	20	161
			2	40	79	34	29	34	216
			3	41	34	93	14	50	232
			4	51	64	32	42	27	216
			5	39	42	62	32	278	453
		%	1	49.1	11.8	14.9	11.8	12.4	100.0
			2	18.5	36.6	15.7	13.4	15.7	100.0
			3	17.7	14.7	40.1	6.0	21.6	100.0
			4	23.6	29.6	14.8	19.4	12.5	100.0
			5	8.6	9.3	13.7	7.1	61.4	100.0
Unselected cases	Original	Count	1	21	9	8	11	9	58
			2	24	29	14	18	23	108
			3	17	15	26	10	21	89
			4	20	11	15	16	13	75
			5	18	15	15	14	110	172
		%	1	36.2	15.5	13.8	19.0	15.5	100.0
			2	22.2	26.9	13.0	16.7	21.3	100.0
			3	19.1	16.9	29.2	11.2	23.6	100.0
			4	26.7	14.7	20.0	21.3	17.3	100.0
			5	10.5	8.7	8.7	8.1	64.0	100.0

a. 44.7% of Selected original grouped cases correctly classified.

b. 40.2% of Unselected original grouped cases correctly classified.

S1.2.2 25pdb low homology dataset + intrinsically disordered proteins (obtained from the *Ideal* database)

Considered protein groups: all-alpha (1) vs all-beta (2) vs alpha/beta (3) vs alpha+beta (4) vs intrinsic_ideal (5)

Supplementary Table S2.1.2.2a
Variables Entered/Removed[a,b,c,d]

	Entered	Removed	Statistic	Between Groups
1	FLWYAEQ		.009	3 and 5
2	FLMWY		.082	3 and 4
3	FYW		.108	2 and 4
4	DEK		.151	2 and 4
5	K		.166	2 and 4
6	D		.172	2 and 4
7	DE		.176	2 and 4
8	E		.178	2 and 4
9	FL		.180	2 and 4
10	AFYWLIV		.181	2 and 4
11	KR		.181	2 and 4
12		DEK	.149	2 and 4
13	AFILMV		.149	2 and 4

At each step. the variable that maximizes the Mahalanobis distance between the two closest groups is entered.

a. Maximum number of steps is 32.

b. Maximum significance of F to enter is .05.

c. Minimum significance of F to remove is .10.

d. F level. tolerance. or VIN insufficient for further computation.

Supplementary Table S1.2.2b
Standardized Canonical Discriminant Function Coefficients

	Function			
	1	2	3	4
AFILMV	-.184	.366	.162	-.513
AFYWLIV	.306	.258	-.701	.015
D	.261	-.206	-.015	-.188
DE	.072	.392	.005	-.009
E	.378	.080	.220	.056
FL	.171	-.164	.365	.267
FLMWY	.260	.404	.398	-.367
FLWYAEQ	-.267	.299	.101	.709
FYW	.319	.052	-.104	.593
K	.368	-.265	-.159	-.200
KR	-.250	.046	.531	.050

Supplementary Table S1.2.2c
Classification Results[a.b]

			Group	Predicted Group Membership 1	2	3	4	5	Total
Selected cases	Original	Count	1	68	24	23	17	11	143
			2	52	85	40	23	27	227
			3	41	33	96	25	30	225
			4	48	48	46	33	17	192
			5	16	35	43	4	290	388
		%	1	47.6	16.8	16.1	11.9	7.7	100.0
			2	22.9	37.4	17.6	10.1	11.9	100.0
			3	18.2	14.7	42.7	11.1	13.3	100.0
			4	25.0	25.0	24.0	17.2	8.9	100.0
			5	4.1	9.0	11.1	1.0	74.7	100.0
Unselected cases	Original	Count	1	30	10	13	13	10	76
			2	14	36	17	14	16	97
			3	17	15	35	10	19	96
			4	21	34	17	17	10	99
			5	9	17	18	2	134	180
		%	1	39.5	13.2	17.1	17.1	13.2	100.0
			2	14.4	37.1	17.5	14.4	16.5	100.0
			3	17.7	15.6	36.5	10.4	19.8	100.0
			4	21.2	34.3	17.2	17.2	10.1	100.0
			5	5.0	9.4	10.0	1.1	74.4	100.0

a. 48.7% of Selected original grouped cases correctly classified.

b. 46.0% of Unselected original grouped cases correctly classified.

S1.2.3 25pdb low homology dataset + intrinsically disordered proteins (obtained from the *Ideal* database)

Considered protein groups: all SCOP (1) vs intrinsic_ideal (5)

Supplementary Table S1.2.3a
Variables in the Equation

		B	S.E.	Wald
Step 1[a]	FYW	-18.740	1.430	171.771
	Constant	2.468	.238	107.207
Step 2[b]	E	-15.602	1.443	116.924
	FYW	-16.989	1.520	124.923
	Constant	4.999	.360	192.464
Step 3[c]	E	-13.923	1.501	86.070
	FYW	-14.427	1.559	85.680
	K	-12.249	1.399	76.696
	Constant	6.361	.425	224.034

Supplementary Table S1.2.3b
Classification Table[c]

Observed			Predicted					
			Selected cases[a]			Unselected cases[b]		
			Group			Group		
			1	5	Percentage Correct	1	5	Percentage Correct
Step 1	Group	1	528	273	65.9	248	106	70.1
		5	118	280	70.4	55	115	67.6
	Overall Percentage				67.4			69.3
Step 2	Group	1	589	212	73.5	256	98	72.3
		5	97	301	75.6	53	117	68.8
	Overall Percentage				74.2			71.2
Step 3	Group	1	614	187	76.7	275	79	77.7
		5	87	311	78.1	49	121	71.2
	Overall Percentage				77.1			75.6

a. Selected cases Random LT 1
b. Unselected cases Random GE 1
c. The cut value is .330

S1.2.4 1189 low homology dataset + intrinsically disordered proteins (obtained from the *DisProt* database)

Considered protein groups: all-alpha (1) vs all-beta (2) vs alpha/beta (3) vs alpha+beta (4) vs intrinsic_disprot (5)

Supplementary Table S1.2.4a
Variables Entered/Removed[a,b,c,d]

	Entered	Removed	Statistic	Between Groups
1	AFYWLIV		.017	2 and 4
2	DEK		.059	2 and 4
3	KR		.118	1 and 3
4	FLWYAEQ		.148	3 and 4
5	KRFYW		.179	3 and 4
6	AFILMV		.227	3 and 4
7	D		.253	2 and 4
8		KRFYW	.185	2 and 4
9	DK		.238	2 and 4
10	R		.257	2 and 4
11	DE		.269	2 and 4
12	FL		.271	2 and 4
13	K		.273	2 and 4
14	E		.273	2 and 4
15		FL	.269	2 and 4
16		DE	.259	2 and 4
17	FLMWY		.259	2 and 4

At each step. the variable that maximizes the Mahalanobis distance between the two closest groups is entered.

a. Maximum number of steps is 32.

b. Maximum significance of F to enter is .05.

c. Minimum significance of F to remove is .10.

d. F level. tolerance. or VIN insufficient for further computation.

Supplementary Table S1.2.4b
Standardized Canonical Discriminant Function Coefficients

	Function			
	1	2	3	4
AFILMV	-.007	.501	-.145	-.159
AFYWLIV	.575	-.321	-.534	.651
D	.365	-.091	.578	.224
DEK	.075	.555	-.092	.114
DK	.146	-.357	-.266	-.376
E	.326	.223	.253	-.039
FLMWY	.238	.443	.186	-.572
FLWYAEQ	-.280	.345	.421	-.022
K	.314	-.102	.175	.382
KR	-.471	.142	.379	.413
R	.174	.197	-.166	.347

Supplementary Table S1.2.4c
Classification Results[a.b]

				Predicted Group Membership					
			Group	1	2	3	4	5	Total
Selected cases	Original	Count	1	52	7	18	7	14	98
			2	19	52	22	12	27	132
			3	43	36	73	23	32	207
			4	22	31	23	16	32	124
			5	46	55	48	24	267	440
		%	1	53.1	7.1	18.4	7.1	14.3	100.0
			2	14.4	39.4	16.7	9.1	20.5	100.0
			3	20.8	17.4	35.3	11.1	15.5	100.0
			4	17.7	25.0	18.5	12.9	25.8	100.0
			5	10.5	12.5	10.9	5.5	60.7	100.0
Unselected cases	Original	Count	1	8	7	2	2	9	28
			2	10	30	8	13	16	77
			3	12	20	28	10	21	91
			4	11	16	6	3	7	43
			5	21	23	21	7	113	185
		%	1	28.6	25.0	7.1	7.1	32.1	100.0
			2	13.0	39.0	10.4	16.9	20.8	100.0
			3	13.2	22.0	30.8	11.0	23.1	100.0
			4	25.6	37.2	14.0	7.0	16.3	100.0
			5	11.4	12.4	11.4	3.8	61.1	100.0

a. 46.0% of Selected original grouped cases correctly classified.

b. 42.9% of Unselected original grouped cases correctly classified.

S1.2.5 1189 low homology dataset + intrinsically disordered proteins (obtained from the *Ideal* database)

Considered protein groups: all-alpha (1) vs all-beta (2) vs alpha/beta (3) vs alpha+beta (4) vs intrinsic_ideal (5)

Supplementary Table S1.2.5a
Variables Entered/Removed[a,b,c,d]

	Entered	Removed	Statistic	Between Groups
1	AFYWLIV		.013	2 and 4
2	DEK		.063	1 and 3
3	K		.116	3 and 4
4	DK		.143	3 and 4
5	AFILMV		.166	3 and 4
6	D		.220	1 and 3
7	FYW		.263	1 and 3
8	FLWYAEQ		.345	1 and 4
9	KR		.360	3 and 4
10	E		.371	3 and 4
11	FL		.373	3 and 4
12	FLMWY		.375	3 and 4
13		FL	.372	3 and 4

At each step. the variable that maximizes the Mahalanobis distance between the two closest groups is entered.

a. Maximum number of steps is 32.

b. Maximum significance of F to enter is .05.

c. Minimum significance of F to remove is .10.

d. F level. tolerance. or VIN insufficient for further computation.

Supplementary Table S1.2.5b
Standardized Canonical Discriminant Function Coefficients

	Function			
	1	2	3	4
AFILMV	-.186	.544	-.061	-.101
AFYWLIV	.487	-.037	-.670	.273
D	.193	-.103	.483	.752
DEK	-.132	.530	.003	.547
DK	.164	-.077	-.399	-.566
E	.361	-.115	.183	-.042
FLMWY	.302	.398	.274	-.357
FLWYAEQ	-.141	.286	.448	.257
FYW	.326	.092	.026	-.318
K	.382	-.082	.242	-.041
KR	-.258	.152	.487	-.381

Supplementary Table S1.2.5c
Classification Results[a,b]

				Predicted Group Membership					
			Group	1	2	3	4	5	Total
Selected cases	Original	Count	1	45	12	16	10	8	91
			2	22	64	19	19	16	140
			3	31	41	75	27	32	206
			4	29	26	21	21	13	110
			5	21	38	25	11	285	380
		%	1	49.5	13.2	17.6	11.0	8.8	100.0
			2	15.7	45.7	13.6	13.6	11.4	100.0
			3	15.0	19.9	36.4	13.1	15.5	100.0
			4	26.4	23.6	19.1	19.1	11.8	100.0
			5	5.5	10.0	6.6	2.9	75.0	100.0
Unselected cases	Original	Count	1	13	5	6	5	6	35
			2	12	27	10	7	13	69
			3	16	15	30	15	16	92
			4	12	22	9	7	7	57
			5	9	17	18	12	132	188
		%	1	37.1	14.3	17.1	14.3	17.1	100.0
			2	17.4	39.1	14.5	10.1	18.8	100.0
			3	17.4	16.3	32.6	16.3	17.4	100.0
			4	21.1	38.6	15.8	12.3	12.3	100.0
			5	4.8	9.0	9.6	6.4	70.2	100.0

a. 52.9% of Selected original grouped cases correctly classified.

b. 47.4% of Unselected original grouped cases correctly classified.

S1.2.6 1189 low homology dataset (subset) + intrinsically disordered proteins (obtained from the *Ideal* database)

Considered protein groups: all SCOP (1) vs intrinsic_ideal (5)

Supplementary Table S1.2.6a
Variables in the Equation

		B	S.E.	Wald
Step 1[a]	E	-15.841	1.404	127.322
	Constant	2.510	.255	96.666
Step 2[b]	E	-13.626	1.452	88.057
	FYW	-13.750	1.556	78.103
	Constant	4.411	.359	151.090
Step 3[c]	E	-13.819	1.493	85.706
	FLMWY	-15.003	2.098	51.121
	FYW	-12.816	1.594	64.625
	Constant	7.389	.590	156.611
Step 4[d]	E	-12.386	1.532	65.392
	FLMWY	-15.858	2.178	52.992
	FYW	-11.534	1.624	50.421
	K	-7.967	1.306	37.203
	Constant	8.453	.649	169.382

a. Variable(s) entered on step 1: E.
b. Variable(s) entered on step 2: FYW.
c. Variable(s) entered on step 3: FLMWY.
d. Variable(s) entered on step 4: K.

Supplementary Table S1.2.6b
Classification Table[c]

			Predicted					
			Selected cases[a]			Unselected cases[b]		
			Group			Group		
	Observed		1	5	Percentage Correct	1	5	Percentage Correct
Step 1	Group	1	393	168	70.1	152	87	63.6
		5	123	268	68.5	57	120	67.8
	Overall Percentage				69.4			65.4
Step 2	Group	1	432	129	77.0	173	66	72.4
		5	124	267	68.3	52	125	70.6
	Overall Percentage				73.4			71.6
Step 3	Group	1	425	136	75.8	181	58	75.7
		5	112	279	71.4	57	120	67.8
	Overall Percentage				73.9			72.4
Step 4	Group	1	443	118	79.0	184	55	77.0
		5	107	284	72.6	50	127	71.8
	Overall Percentage				76.4			74.8

a. Selected cases Random LT 1
b. Unselected cases Random GE 1
c. The cut value is .450

S1.3 Classification of transmembrane proteins (subset obtained from http://pdbtm.enzim.hu)

Considered protein groups: beta-sheets (1) vs alpha-helixes (2)

Supplementary Table S1.3a
Variables in the Equation

		B	S.E.	Wald
Step 1[a]	AFILMV	41.917	4.503	86.654
	Constant	-9.566	1.080	78.423
Step 2[b]	AFILMV	40.646	4.715	47.995
	DE	-9.055	3.822	45.849
	Constant	7.844	1.297	91.542
Step 3[c]	AFILMV	38.233	5.181	45.394
	DE	-8.473	4.209	32.816
	FYW	-11.597	3.101	26.435
	Constant	-5.142	1.493	32.589
Step 4[d]	AFILMV	38.771	5.296	28.464
	DE	-9.131	3.339	27.598
	DK	-11.547	4.284	24.447
	FYW	1.704	1.087	6.416
	Constant	-5.447	1.793	15.832

a. Variable(s) entered on step 1: AFILMV.

b. Variable(s) entered on step 2: DE.

c. Variable(s) entered on step 3: FYW.

d. Variable(s) entered on step 4: DK.

Supplementary Table S1.3b
Classification Table[c]

			Predicted					
			Selected cases[a]			Unselected cases[b]		
			Group			Group		
Observed			1.00	2.00	Percentage Correct	1.00	2.00	Percentage Correct
Step 1	Group	1.00	385	46	89.3	167	16	91.3
		2.00	68	345	83.5	27	148	85.6
	Overall Percentage				86.5			88.0
Step 2	Group	1.00	377	54	87.5	160	23	87.4
		2.00	44	369	89.3	12	163	93.1
	Overall Percentage				88.4			90.2
Step 3	Group	1.00	377	54	87.5	166	17	90.7
		2.00	44	369	89.3	12	163	93.1
	Overall Percentage				88.4			91.9
Step 4	Group	1.00	389	42	90.3	166	17	90.7
		2.00	47	366	88.6	19	156	89.1
	Overall Percentage				89.5			89.9

a. Selected cases Approximately 70% of the cases (SAMPLE) EQ 1

b. Unselected cases Approximately 70% of the cases (SAMPLE) NE 1

c. The cut value is 0.630

S2 Classification by localization properties

S2.1 Low-homology data obtained from UniProtKB/Swiss-Prot database

S2.1.1 Prediction of membrane associated proteins

Considered protein groups: Cytosolic and nuclear (1) vs Membrane (2)

Supplementary Table S2.1.1a
Variables in the Equation

		B	S.E.	Wald
Step 1[a]	DEK	-29.934	.172	30273.101
	Constant	4.862	.033	21966.900
Step 2[b]	DEK	-28.542	.177	25904.514
	FLMWY	16.261	.204	6360.008
	Constant	.985	.056	304.305
Step 3[c]	DEK	-25.293	.187	18370.053
	FLMWY	15.208	.206	5453.942
	KR	-7.243	.149	2358.325
	Constant	1.758	.059	883.972
Step 4[d]	DEK	-26.279	.196	17972.201
	FLMWY	10.580	.220	2305.803
	KR	-13.732	.185	5514.808
	KRFYW	12.848	.189	4637.805
	Constant	.941	.062	232.820

Supplementary Table S2.1.1b
Classification Table[c]

			Predicted					
			Selected cases[a]			Unselected cases[b]		
			membr			membr		
	Observed		1	2	Percentage Correct	1	2	Percentage Correct
Step 1	membr	1	80716	22864	77.9	34531	9768	77.9
		2	11335	30848	73.1	4968	13093	72.5
	Overall Percentage				76.5			76.4
Step 2	membr	1	82468	21112	79.6	35185	9114	79.4
		2	10997	31186	73.9	4814	13247	73.3
	Overall Percentage				78.0			77.7
Step 3	membr	1	82967	20613	80.1	35382	8917	79.9
		2	10915	31268	74.1	4798	13263	73.4
	Overall Percentage				78.4			78.0
Step 4	membr	1	84635	18945	81.7	36022	8277	81.3
		2	10198	31985	75.8	4417	13644	75.5
	Overall Percentage				80.0			79.6

a. Selected cases random LT 1
b. Unselected cases random GE 1
c. The cut value is .290

S2.1.2 Classification of membrane associated proteins

Considered protein groups: monotopic (1) vs bi- and polytopic transmembrane (2)

Supplementary Table S2.1.2a
Variables in the Equation

		B	S.E.	Wald
Step 1[a]	DEK	-13.657	2.927	21.766
	Constant	3.167	.580	29.796
Step 2[b]	DEK	-30.160	4.642	42.212
	DK	26.111	4.202	38.612
	Constant	2.456	.709	12.001
Step 3[c]	DEK	-28.270	4.730	35.719
	DK	28.037	4.447	39.756
	KRFYW	-12.628	3.226	15.320
	Constant	5.008	.990	25.595
Step 4[d]	DEK	-27.502	4.905	31.440
	DK	27.319	4.592	35.388
	FYW	12.625	4.392	8.262
	KRFYW	-13.899	3.403	16.681
	Constant	3.136	1.164	7.258

a. Variable(s) entered on step 1: DEK.

b. Variable(s) entered on step 2: DK.

c. Variable(s) entered on step 3: KRFYW.

d. Variable(s) entered on step 4: FYW.

Supplementary Table S2.1.2b
Classification Table[c]

Observed			Predicted					
			Selected cases[a]			Unselected cases[b]		
			Group			Group		
			1	2	Percentage Correct	1	2	Percentage Correct
Step 1	Group	1	70	27	72.2	36	18	66.7
		2	54	130	70.7	18	55	75.3
	Overall Percentage				71.2			71.7
Step 2	Group	1	85	12	87.6	42	12	77.8
		2	54	130	70.7	18	55	75.3
	Overall Percentage				76.5			76.4
Step 3	Group	1	86	11	88.7	45	9	83.3
		2	50	134	72.8	14	59	80.8
	Overall Percentage				78.3			81.9
Step 4	Group	1	84	13	86.6	45	9	83.3
		2	47	137	74.5	12	61	83.6
	Overall Percentage				78.6			83.5

a. Selected cases Random LT 1

b. Unselected cases Random GE 1

c. The cut value is .630

S2.1.3 Classification of polytopic transmembrane proteins

Considered protein groups: single-pass (3) vs multi-pass (4)

Supplementary Table S2.1.3a
Variables in the Equation

		B	S.E.	Wald
Step 1[a]	FLMWY	34.510	.401	7417.804
	Constant	-7.024	.091	5915.779
Step 2[b]	DEK	-18.686	.302	3817.378
	FLMWY	31.269	.419	5572.443
	Constant	-3.070	.107	827.093
Step 3[c]	DEK	-15.336	.319	2304.073
	FLMWY	30.302	.426	5068.612
	KR	-9.176	.273	1126.961
	Constant	-2.076	.112	346.214
Step 4[d]	AFILMV	16.360	.481	1157.086
	DEK	-14.512	.326	1976.339
	FLMWY	24.589	.449	2996.424
	KR	-9.609	.280	1176.860
	Constant	-4.895	.142	1179.912

Supplementary Table S2.1.3b
Classification Table[d]

			Predicted					
			Selected cases[a]			Unselected cases[b,.c]		
			passes			passes		
Observed			3	4	Percentage Correct	3	4	Percentage Correct
Step 1	passes	3	8312	3779	68.7	3605	1629	68.9
		4	7045	23047	76.6	2997	9830	76.6
	Overall Percentage				74.3			74.4
Step 2	passes	3	9276	2815	76.7	4089	1145	78.1
		4	6576	23516	78.1	2796	10031	78.2
	Overall Percentage				77.7			78.2
Step 3	passes	3	9540	2551	78.9	4187	1047	80.0
		4	6426	23666	78.6	2727	10100	78.7
	Overall Percentage				78.7			79.1
Step 4	passes	3	9663	2428	79.9	4249	985	81.2
		4	6137	23955	79.6	2591	10236	79.8
	Overall Percentage				79.7			80.2

a. Selected cases random LT 1

b. Unselected cases random GE 1

c. Some of the Selected cases are not classified due to either missing values in the independent variables or categorical variables with values out of the range of the Selected cases.

d. The cut value is .700

S2.1.4 Classification of non-membrane proteins

Considered protein groups: cytoplasm (1) vs nucleus (2)

Supplementary Table S2.1.4a
Variables in the Equation

		B	S.E.	Wald
Step 1[a]	AFYWLIV	-31.580	.300	11104.256
	Constant	7.716	.084	8430.788
Step 2[b]	AFYWLIV	-31.484	.307	10504.747
	DE	-13.516	.211	4087.226
	Constant	10.035	.096	11022.017
Step 3[c]	AFYWLIV	-32.709	.314	10822.148
	DE	-12.734	.216	3486.247
	K	-6.077	.152	1600.056
	Constant	11.217	.103	11970.818
Step 4[d]	AFYWLIV	-33.606	.321	10967.106
	DE	-13.324	.220	3678.919
	K	-5.727	.156	1354.010
	KRFYW	6.206	.171	1318.969
	Constant	9.996	.107	8704.538

a. Variable(s) entered on step 1: AFYWLIV.
b. Variable(s) entered on step 2: DE.
c. Variable(s) entered on step 3: K.
d. Variable(s) entered on step 4: KRFYW.

Supplementary Table S2.1.4b
Classification Table[d]

		Predicted					
		Selected cases[a]			Unselected cases[b,c]		
		nucleus			nucleus		
	Observed	1	2	Percentage Correct	1	2	Percentage Correct
Step 1	nucleus 1	57644	22159	72.2	24572	9504	72.1
	2	8631	15146	63.7	3747	6476	63.3
	Overall Percentage			70.3			70.1
Step 2	nucleus 1	58207	21596	72.9	24921	9155	73.1
	2	7785	15992	67.3	3345	6878	67.3
	Overall Percentage			71.6			71.8
Step 3	nucleus 1	58702	21101	73.6	25072	9004	73.6
	2	7467	16310	68.6	3176	7047	68.9
	Overall Percentage			72.4			72.5
Step 4	nucleus 1	58642	21161	73.5	25043	9033	73.5
	2	7013	16764	70.5	3017	7206	70.5
	Overall Percentage			72.8			72.8

a. Selected cases random LT 1

b. Unselected cases random GE 1

c. Some of the Selected cases are not classified due to either missing values in the independent variables or categorical variables with values out of the range of the Selected cases.

d. The cut value is .230

S2.2 High homology data from pathogenic *Firmicutes* and *Proteobacteria* (obtained from http://www.mgc.ac.cn/VFs/main.htm)

S2.2.1 Prediction of membrane proteins

Considered protein groups: DNA-binding (1) vs membrane-associated (3) proteins from *Proteobacteria*

Supplementary Table S2.2.1a
Variables in the Equation

		B	S.E.	Wald
Step 1[a]	FL	-33.098	4.062	66.406
	Constant	7.092	.819	74.979
Step 2[b]	FL	-44.385	5.171	73.688
	KR	-22.106	3.625	37.194
	Constant	13.037	1.460	79.766
Step 3[c]	FL	-42.300	6.077	48.453
	KR	-34.163	4.832	49.993
	KRFYW	28.459	4.178	46.406
	Constant	7.702	1.725	19.931
Step 4[d]	FL	-56.028	8.585	42.592
	KR	-36.161	5.634	41.197
	KRFYW	36.480	5.297	47.425
	R	-21.917	4.171	27.608
	Constant	11.232	2.352	22.802

a. Variable(s) entered on step 1: FL.
b. Variable(s) entered on step 2: KR.
c. Variable(s) entered on step 3: KRFYW.
d. Variable(s) entered on step 4: R.
e. Variable(s) entered on step 5: M.

Supplementary Table S2.2.1b
Classification Table[d]

			Predicted					
			Selected cases[a]			Unselected cases[b,c]		
			Group13			Group13		
	Observed		1	3	Percentage Correct	1	3	Percentage Correct
Step 1	Group13	1	90	23	79.6	45	14	76.3
		3	48	164	77.4	23	74	76.3
	Overall Percentage				78.2			76.3
Step 2	Group13	1	91	22	80.5	45	14	76.3
		3	41	171	80.7	24	73	75.3
	Overall Percentage				80.6			75.6
Step 3	Group13	1	101	12	89.4	52	7	88.1
		3	27	185	87.3	14	83	85.6
	Overall Percentage				88.0			86.5
Step 4	Group13	1	108	5	95.6	54	5	91.5
		3	21	191	90.1	8	89	91.8
	Overall Percentage				92.0			91.7

a. Selected cases Random LT 1
b. Unselected cases Random GE 1
c. Some of the Selected cases are not classified due to either missing values in the independent variables or categorical variables with values out of the range of the Selected cases.
d. The cut value is .640

S2.2.2 Prediction of membrane proteins (obtained from http://www.mgc.ac.cn/VFs/main.htm)

Considered protein groups: DNA-binding (2) vs membrane-associated (4) proteins from pathogenic *Firmicutes*

Supplementary Table S2.2.2a
Variables in the Equation

		B	S.E.	Wald
Step 1[a]	DEK	-27.558	5.983	21.218
	Constant	7.868	1.496	27.680
Step 2[b]	AFYWLIV	-45.739	11.385	16.141
	DEK	-28.498	6.103	21.803
	Constant	21.770	3.980	29.913
Step 3[c]	AFYWLIV	-49.787	12.841	15.033
	DEK	-44.350	8.890	24.886
	DK	37.612	10.061	13.976
	Constant	20.365	4.277	22.671
Step 4[d]	AFYWLIV	-47.108	13.888	11.505
	DEK	-52.605	10.392	25.627
	DK	38.231	10.063	14.435
	M	-14.038	5.237	7.184
	Constant	22.247	4.652	22.873

a. Variable(s) entered on step 1: DEK.
b. Variable(s) entered on step 2: AFYWLIV.
c. Variable(s) entered on step 3: DK.
d. Variable(s) entered on step 4: M.

Supplementary Table S2.2.2b
Classification Table[d]

Observed			Predicted					
			Selected cases[a]			Unselected cases[b..c]		
			Group24			Group24		
			2	4	Percentage Correct	2	4	Percentage Correct
Step 1	Group24	2	23	10	69.7	13	2	86.7
		4	51	87	63.0	21	41	66.1
	Overall Percentage				64.3			70.1
Step 2	Group24	2	25	8	75.8	9	6	60.0
		4	28	110	79.7	11	51	82.3
	Overall Percentage				78.9			77.9
Step 3	Group24	2	27	6	81.8	10	5	66.7
		4	28	110	79.7	13	49	79.0
	Overall Percentage				80.1			76.6
Step 4	Group24	2	28	5	84.8	11	4	73.3
		4	21	117	84.8	14	48	77.4
	Overall Percentage				84.8			76.6

a. Selected cases Random LT 1

b. Unselected cases Random GE 1

c. Some of the Selected cases are not classified due to either missing values in the independent variables or categorical variables with values out of the range of the Selected cases.

d. The cut value is .810

Supplementary S2.2.3 Prediction of membrane proteins by host organism

Considered protein groups: membrane-associated proteins from pathogenic *Proteobacteria* (3) and *Firmicutes* (4)

Supplementary Table S2.2.3a
Variables in the Equation

		B	S.E.	Wald
Step 1[a]	AFILMV	21.921	4.495	23.786
	Constant	-5.823	1.098	28.146
Step 2[b]	AFILMV	25.525	4.704	29.445
	FLWYAEQ	-21.305	5.461	15.218
	Constant	-.643	1.670	.148
Step 3[c]	AFILMV	32.545	5.168	39.657
	DEK	12.485	2.839	19.339
	FLWYAEQ	-19.235	5.755	11.170
	Constant	-5.287	2.058	6.600

a. Variable(s) entered on step 1: AFILMV.

b. Variable(s) entered on step 2: DEK.

c. Variable(s) entered on step 3: FLWYAEQ.

Supplementary Table S2.2.3b

Classification Table[d]

			Predicted					
			Selected cases[a]			Unselected cases[b..c]		
			Group34			Group34		
	Observed		3	4	Percentage Correct	3	4	Percentage Correct
Step 1	Group34	3	176	53	76.9	62	18	77.5
		4	68	67	49.6	34	31	47.7
	Overall Percentage				66.8			64.1
Step 2	Group34	3	183	34	84.3	74	18	80.4
		4	27	113	80.7	16	44	73.3
	Overall Percentage				82.9			77.6
Step 3	Group34	3	194	23	89.4	79	13	85.9
		4	20	120	85.7	14	46	76.7
	Overall Percentage				88.0			82.2

a. Selected cases Random LT 1

b. Unselected cases Random GE 1

c. Some of the Selected cases are not classified due to either missing values in the independent variables or categorical variables with values out of the range of the Selected cases.

d. The cut value is .41

S3 Classification by functional properties

S3.1 Classification of non-membrane proteins obtained from UniProtKB/Swiss-Prot database

S3.1.1 General functional classification

Considered protein groups: cytoskeletons (1) vs enzymes (2) vs transcription factors (3)

Supplementary Table S3.1.1a
Variables Entered/Removed[a.b.c.d]

	Entered	Statistic	Between Groups
1	KR	.040	2 and 3
2	FLMWY	.202	2 and 3
3	AFYWLIV	.271	1 and 3
4	FYW	.321	1 and 3
5	DEK	.364	1 and 3
6	FL	.378	1 and 3
7	K	.396	1 and 3
8	FLWYAEQ	.411	1 and 3
9	DE	.421	1 and 3
10	D	.427	1 and 3
11	AFILMV	.431	1 and 3
12	R	.435	1 and 3
13	E	.439	1 and 3
14	M	.441	1 and 3
15	DK	.443	1 and 3
16	KRFYW	.444	1 and 3

At each step. the variable that maximizes the Mahalanobis distance between the two closest groups is entered.

a. Maximum number of steps is 32.

b. Maximum significance of F to enter is .05.

c. Minimum significance of F to remove is .10.

d. F level. tolerance. or VIN insufficient for further computation.

Supplementary Table S3.1.1b
Standardized Canonical Discriminant Function Coefficients

	Function	
	1	2
AFILMV	.126	-.143
AFYWLIV	.770	.090
D	.069	-.143
DE	.214	.224
DEK	-.211	.230
DK	.068	.084
E	.036	-.103
FL	-.074	-.221
FLMWY	.098	.545
FLWYAEQ	-.157	.201
FYW	.119	-.363
K	.153	.226
KR	-.330	.387
KRFYW	.170	.037
M	.130	.071
R	.054	.105

Supplementary Table S3.1.1c
Classification Results[a,b]

				Predicted Group Membership			Total
			Group	1	2	3	
Selected cases	Original	Count	1	2236	991	956	4183
			2	8185	20174	6371	34730
			3	4489	3983	6148	14620
		%	1	53.5	23.7	22.9	100.0
			2	23.6	58.1	18.3	100.0
			3	30.7	27.2	42.1	100.0
Unselected cases	Original	Count	1	858	436	446	1740
			2	3526	8683	2697	14906
			3	1868	1611	2649	6128
		%	1	49.3	25.1	25.6	100.0
			2	23.7	58.3	18.1	100.0
			3	30.5	26.3	43.2	100.0

a. 53.3% of Selected original grouped cases correctly classified.

b. 53.5% of Unselected original grouped cases correctly classified.

S3.1.2 Prediction of cytoskeleton proteins among non-membrane proteins (Selected cases)

Considered protein groups: cytoskeletons (1) vs others (2)

Supplementary Table S3.1.2a
Variables in the Equation

		B	S.E.	Wald
Step 1[a]	KR	-9.584	.446	461.694
	Constant	1.804	.086	436.067
Step 2[b]	AFYWLIV	11.562	.863	179.290
	KR	-8.976	.453	391.824
	Constant	-1.596	.266	36.112
Step 3[c]	AFYWLIV	11.790	.867	184.834
	FYW	5.428	.458	140.488
	KR	-7.962	.462	297.511
	Constant	-2.696	.283	90.693
Step 4[d]	AFYWLIV	12.229	.874	195.953
	DEK	-5.834	.627	86.475
	FYW	5.680	.461	151.889
	KR	-6.210	.497	155.867
	Constant	-1.940	.295	43.352
Step 5[e]	AFYWLIV	15.469	.967	256.001
	DEK	-5.794	.630	84.552
	FLMWY	-6.064	.723	70.345
	FYW	6.313	.474	177.785
	KR	-6.247	.500	156.021
	Constant	-1.655	.298	30.757
Step 6[f]	AFYWLIV	16.310	.982	276.074
	DEK	-6.834	.654	109.096
	E	3.279	.489	45.033
	FLMWY	-6.037	.725	69.247
	FYW	5.675	.483	138.065
	KR	-5.897	.504	136.734
	Constant	-2.179	.310	49.390

a. Variable(s) entered on step 1: KR.
b. Variable(s) entered on step 2: AFYWLIV.
c. Variable(s) entered on step 3: FYW.
d. Variable(s) entered on step 4: DEK.
e. Variable(s) entered on step 5: FLMWY.
f. Variable(s) entered on step 6: E.

Supplementary Table S3.1.2b
Classification Table[c]

Observed			Predicted: Selected cases[a] Group12 1	2	Percentage Correct	Unselected cases[b] Group12 1	2	Percentage Correct
Step 1	Group12	1	2466	1696	59.3	1040	721	59.1
		2	1695	2520	59.8	700	1118	61.5
	Overall Percentage				59.5			60.3
Step 2	Group12	1	2467	1695	59.3	1051	710	59.7
		2	1521	2694	63.9	655	1163	64.0
	Overall Percentage				61.6			61.9
Step 3	Group12	1	2520	1642	60.5	1069	692	60.7
		2	1513	2702	64.1	656	1162	63.9
	Overall Percentage				62.3			62.3
Step 4	Group12	1	2537	1625	61.0	1080	681	61.3
		2	1478	2737	64.9	663	1155	63.5
	Overall Percentage				63.0			62.4
Step 5	Group12	1	2599	1563	62.4	1094	667	62.1
		2	1506	2709	64.3	664	1154	63.5
	Overall Percentage				63.4			62.8
Step 6	Group12	1	2660	1502	63.9	1113	648	63.2
		2	1532	2683	63.7	641	1177	64.7
	Overall Percentage				63.8			64.0

a. Selected cases Random LT 1

b. Unselected cases Random GE 1

c. The cut value is .500

S3.1.3 Prediction of cytosolic non-cytoskeletal proteins by function

Considered protein groups: enzymes (2) vs transcription factors (3)

Supplementary Table S3.1.3a
Variables in the Equation

		B	S.E.	Wald
Step 1[a]	AFYWLIV	-23.496	.375	3930.340
	Constant	5.810	.106	2983.677
Step 2[b]	AFYWLIV	-23.960	.378	4019.160
	K	-3.168	.175	327.401
	Constant	6.442	.113	3245.032
Step 3[c]	AFYWLIV	-23.785	.380	3913.074
	K	-3.245	.177	335.107
	KR	3.070	.201	232.143
	Constant	5.866	.119	2425.944
Step 4[d]	AFYWLIV	-23.612	.381	3843.073
	DE	-3.839	.255	225.865
	K	-2.829	.179	250.810
	KR	3.288	.203	261.590
	Constant	6.362	.124	2618.463
Step 5[e]	AFYWLIV	-22.614	.387	3420.574
	DE	-3.953	.256	238.362
	K	-3.278	.182	323.511
	KR	5.278	.252	438.172
	KRFYW	-3.562	.261	185.941
	Constant	6.681	.127	2768.713

a. Variable(s) entered on step 1: AFYWLIV.
b. Variable(s) entered on step 2: K.
c. Variable(s) entered on step 3: KR.
d. Variable(s) entered on step 4: DE.
e. Variable(s) entered on step 5: KRFYW.

Supplementary Table S3.1.3b

Classification Table[d]

Observed			Predicted					
			Selected cases[a]			Unselected cases[b..c]		
			Group23			Group23		
			2	3	Percentage Correct	2	3	Percentage Correct
Step 1	Group23	2	23555	11182	67.8	10131	4768	68.0
		3	5798	8652	59.9	2548	3750	59.5
	Overall Percentage				65.5			65.5
Step 2	Group23	2	23559	11178	67.8	10137	4762	68.0
		3	5749	8701	60.2	2498	3800	60.3
	Overall Percentage				65.6			65.7
Step 3	Group23	2	23409	11328	67.4	10041	4858	67.4
		3	5574	8876	61.4	2409	3889	61.7
	Overall Percentage				65.6			65.7
Step 4	Group23	2	23488	11249	67.6	10105	4794	67.8
		3	5630	8820	61.0	2448	3850	61.1
	Overall Percentage				65.7			65.8
Step 5	Group23	2	23598	11139	67.9	10100	4799	67.8
		3	5623	8827	61.1	2438	3860	61.3
	Overall Percentage				65.9			65.9

a. Selected cases Random LT 1

b. Unselected cases Random GE 1

c. Some of the Selected cases are not classified due to either missing values in the independent variables or categorical variables with values out of the range of the Selected cases.

d. The cut value is .290

S3.2 Classification of membrane proteins obtained from RCSB PDB database

Considered protein groups: ATP-binding (1) vs Transporters (2) vs G-protein receptors (3) vs Photosynthetic (4) vs Rhodopsines (5)

Supplementary Table S3.2a
Variables Entered/Removed[a.b.c.d]

	Entered	Removed	Statistic	Between Groups
1	KR		.041	2 and 4
2	FLWYAEQ		.511	1 and 3
3	DEK		.943	3 and 4
4	FLMWY		1.296	3 and 4
5	K		1.657	2 and 3
6	KRFYW		2.102	1 and 3
7	R		2.473	2 and 3
8	DE		2.700	2 and 3
9	D		2.921	2 and 3
10	AFYWLIV		3.045	2 and 3
11	DK		3.168	2 and 3
12		D	2.800	2 and 3
13	FL		2.854	2 and 3
14		FLMWY	2.750	2 and 3
15	E		2.751	2 and 3
16	FYW		2.753	2 and 3
17	AFILMV		2.754	2 and 3

At each step. the variable that maximizes the Mahalanobis distance between the two closest groups is entered.

a. Maximum number of steps is 32.

b. Maximum significance of F to enter is .05.

c. Minimum significance of F to remove is .10.

d. F level. tolerance. or VIN insufficient for further computation.

Supplementary Table S3.2b
Standardized Canonical Discriminant Function Coefficients

	Function			
	1	2	3	4
AFILMV	.386	-.146	.325	.184
AFYWLIV	-.347	.180	-.345	-.342
DE	-.290	-.342	-.361	-.376
DEK	.174	-.479	.622	.188
DK	-.458	.455	.048	-.347
E	.444	.128	.040	.084
FL	-.571	-.501	-.178	.488
FLWYAEQ	.707	.252	.287	-.370
FYW	.358	.113	-.193	.104
K	.615	.148	.590	-.318
KR	-.259	.095	.423	-.210
KRFYW	-.242	.435	.187	.571
R	.119	-.146	-.426	.404

Supplementary Table S3.2c
Classification Results[a.b]

				Predicted Group Membership					
			Group	1	2	3	4	5	Total
Selected cases	Original	Count	1	31	5	3	2	2	43
			2	4	39	15	3	2	63
			3	5	5	21	0	0	31
			4	4	1	0	28	0	33
			5	0	1	1	2	28	32
		%	1	72.1	11.6	7.0	4.7	4.7	100.0
			2	6.3	61.9	23.8	4.8	3.2	100.0
			3	16.1	16.1	67.7	.0	.0	100.0
			4	12.1	3.0	.0	84.8	.0	100.0
			5	.0	3.1	3.1	6.3	87.5	100.0
Unselected cases	Original	Count	1	14	0	0	1	0	15
			2	1	11	5	3	2	22
			3	0	1	7	1	0	9
			4	2	0	0	10	0	12
			5	0	0	0	1	7	8
		%	1	93.3	.0	.0	6.7	.0	100.0
			2	4.5	50.0	22.7	13.6	9.1	100.0
			3	.0	11.1	77.8	11.1	.0	100.0
			4	16.7	.0	.0	83.3	.0	100.0
			5	.0	.0	.0	12.5	87.5	100.0

a. 72.8% of Selected original grouped cases correctly classified.

b. 74.2% of Unselected original grouped cases correctly classified.

S3.3 Classification of membrane proteins (Selected example)

Considered protein groups: ATP-binding (1) vs Transporters (2)

Supplementary Table S3.3a
Variables in the Equation

		B	S.E.	Wald
Step 1[a]	KRFYW	-26.290	6.316	17.326
	Constant	6.338	1.519	17.417
Step 2[b]	DK	-14.877	4.392	11.475
	KRFYW	-34.823	7.541	21.324
	Constant	10.879	2.271	22.940
Step 3[c]	AFYWLIV	23.761	10.451	5.169
	DK	-17.032	4.909	12.036
	KRFYW	-34.930	7.715	20.500
	Constant	4.202	3.517	1.428

a. Variable(s) entered on step 1: KRFYW.

b. Variable(s) entered on step 2: DK.

c. Variable(s) entered on step 3: AFYWLIV.

Supplementary Table S3.3b

Classification Table[c]

			Predicted					
			Selected cases[a]			Unselected cases[b]		
			Group			Group		
Observed			1	2	Percentage Correct	1	2	Percentage Correct
Step 1	Group	1	31	10	75.6	15	2	88.2
		2	22	30	57.7	16	17	51.5
	Overall Percentage				65.6			64.0
Step 2	Group	1	31	10	75.6	14	3	82.4
		2	13	39	75.0	10	23	69.7
	Overall Percentage				75.3			74.0
Step 3	Group	1	33	8	80.5	15	2	88.2
		2	13	39	75.0	8	25	75.8
	Overall Percentage				77.4			80.0

a. Selected cases Approximately 70% of the cases (SAMPLE) EQ 1

b. Unselected cases Approximately 70% of the cases (SAMPLE) NE 1

c. The cut value is .600

S3.4 Classification of membrane proteins (Selected example for transmembrane proteins of *Firmicutes* except *Heliobacteria*, no photosynthetic, no ATP-binding)

Considered protein groups: Transporters (2) vs Sensors (3)

Supplementary Table S3.4a

Variables in the Equation

		B	S.E.	Wald
Step 1[a]	KRFYW	33.441	7.667	19.023
	Constant	-8.850	1.954	20.521

a. Variable(s) entered on step 1: KRFYW.

Supplementary Table S3.4b
Classification Table[c]

Observed			Predicted					
			Selected cases[a]			Unselected cases[b]		
			Group			Group		
			2	3	Percentage Correct	2	3	Percentage Correct
Step 1	Group	2	47	15	75.8	18	5	78.3
		3	8	22	73.3	2	8	80.0
	Overall Percentage				75.0			78.8

a. Selected cases Approximately 70% of the cases (SAMPLE) EQ 1

b. Unselected cases Approximately 70% of the cases (SAMPLE) NE 1

c. The cut value is .330
